# Chemosensory protein regulates the behavioural response of *Frankliniella intonsa* and *Frankliniella occidentalis* to tomato zonate spot virus–Infected pepper (*Capsicum annuum*)

**DOI:** 10.1371/journal.ppat.1011380

**Published:** 2023-05-08

**Authors:** Heng Li, Yixin Chen, Chengcong Lu, Houjun Tian, Shuo Lin, Liang Wang, Tingting Linghu, Xue Zheng, Hui Wei, Xiaojing Fan, Yong Chen

**Affiliations:** 1 State Key Laboratory of Ecological Pest Control for Fujian and Taiwan Crops, Institute of Plant Protection, Fujian Academy of Agricultural Sciences, Fuzhou, China; 2 Fujian Key Laboratory for Monitoring and Integrated Management of Crop Pests, Fuzhou Scientific Observing and Experimental Station of Crop Pests of Ministry of Agriculture, Fujian Engineering Research Center for Green Pest Management, Fuzhou, China; 3 Fujian Agriculture and Forestry University, Fuzhou, China; 4 Institute of Biotechnology and Germplasm Resources, Yunnan Academy of Agricultural Sciences, Kunming, China; University of Cambridge, UNITED STATES

## Abstract

Many herbivorous insects rely on plant volatiles to locate their host plants. Vector-borne viral infections induce changes in plant volatiles, which render infected plants more attractive to insect vectors. However, the detailed mechanisms underlying the olfactory responses of insect vectors induced by the volatiles produced by virus-infected plants are poorly understood. Here, we show that volatiles emitted by pepper (*Capsicum annuum*) plants infected with tomato zonate spot virus (TZSV), particularly the volatile *cis*-3-hexenal, which is recognized by chemosensory protein 1 of the thrips *Frankliniella intonsa* (FintCSP1), are more attractive to *F*. *intonsa* than the volatiles emitted by non-infected pepper plants. FintCSP1 is highly abundant in the antenna of *F*. *intonsa*. Silencing of *FintCSP1* significantly decreased electroantennogram responses of *F*. *intonsa* antennae to *cis*-3-hexenal and impaired thrips’ responses to TZSV-infected pepper plants and *cis*-3-hexenal, as assessed using a Y-tube olfactometer. Three-dimensional model predictions indicated that FintCSP1 consists of seven α-helixes and two disulfide bridges. Molecular docking analysis suggested that *cis*-3-hexenal is positioned deep inside the binding pocket of FintCSP1 and binds to residues of the protein. We combined site-directed mutagenesis and fluorescence binding assays and identified three hydrophilic residues, Lys26, Thr28, and Glu67, of FintCSP1 as being critical for *cis*-3-hexenal binding. Furthermore, CSP of *F*. *occidentalis* (FoccCSP) is also a key olfactory protein involved in modulating the behaviour of *F*. *occidentalis* to TZSV-infected pepper. This study revealed the specific binding characteristics of CSPs to *cis*-3-hexenal and confirmed the general hypothesis that virus infections induce changes in host volatiles, which can be recognized by the olfactory proteins of the insect vector to enhance vector attraction and this may facilitate viral spread and transmission.

## Author summary

Arboviruses rely on insect vectors for their transmission, and promote disease transmission by influencing insect behaviour after infecting plant hosts. However, the detailed olfactory responses involved in this process are poorly understood. Here, we demonstrate that a persistent plant virus induces the production of a unique volatile in its host plant that is recognized by a key olfactory protein in its insect vector. Furthermore, this unique volatile induced by virus infection fits deep inside the binding pocket of its cognate olfactory protein, where it interacts with several hydrophilic residues. This work reveals that a plant virus can manipulate the behaviour of its insect vectors, suggesting the modification of insect behaviour by chemicals as a management program to control the spread of plant viruses.

## Introduction

Most plant viruses that threaten agricultural crops rely on insect vectors for their transmission [1–3]. There are three types of insect transmission of plant viruses: nonpersistent transmission, semipersistent transmission, and persistent transmission [1,3]. In persistent transmission, insects retain the acquired virus for long periods (days to weeks), transmitting the virus often throughout the vector’s lifespan [1,3]. A large body of literature has documented that viruses improve the nutrient composition of their host plants, which consequently increases the population growth parameters of virus vectors. Sometimes, viruses alter the profile of volatile compounds emitted by their hosts, making infected plants more attractive to insect vectors, thereby bringing more insect vectors to infected plants and thus promoting viral spread [4–8]. In certain host-virus-vector interaction, virus-induced volatile organic compounds (VOCs) alter emission of host which does not make the host plant more attractive to insect vectors [9]. Moreover, aphid vectors are initially more attracted to virus-infected plant, but they are then subsequently deterred from feeding on the host plant after a period of time [10,11]. Although virus-induced plant VOCs help insect vectors locate host plants [12–14], how VOCs modify insect behaviour is poorly understood.

The insect olfactory system can identify minute amounts of volatile semiochemicals released from host plants or environments, which play an important role in mediating insect behavioural responses, such as seeking food, selecting oviposition sites, or avoiding enemies [15,16]. Olfaction in insects relies on olfactory proteins, including chemosensory proteins (CSPs), odorant binding proteins (OBPs), odorant receptors (ORs), ionotropic receptors, sensory neuron membrane proteins, and odorant degrading enzymes, which are all present in the sensillar lymph of the antennae, maxillary palps, legs, and other chemosensory structures [17–19]. CSPs are small, soluble polypeptides that are highly abundant in chemosensory organs of insects [16]. In insects, CSPs function in chemoreception, pheromone recognition, embryo development, regeneration of amputated limbs, solubilization of nutrients, insecticide resistance, and immune responses [16]. The best studied function of CSPs is in chemical communication. To be recognized, the odorous molecules must first pass through the pores or slits in the surface of the insect’s antennae or maxillary palps to sensory lymphatic fluid, after which they bind to OBPs or CSPs to form a complex and then are transferred to the dendritic processes of the sensory neurons to finally activate membrane-bound ORs [17,20,21]. In addition, odorous molecules directly interact with ORs to initiate an olfactory-related signal transduction cascade. Thus, OBPs and CSPs commonly serve as ligand selectors, transporters, and solubilizers to trigger a signal or as deactivators once the olfactory signal has been perceived [17,22,23]. However, how these proteins function in insect vectors to locate virus-infected host plants have not been elucidated.

Some insect-borne plant virus-induced alterations in the emissions of host VOCs influence the behaviour of insect vectors and promote local virus transmission [24,25]. In this study, we selected tomato zonate spot virus (TZSV), a newly identified virus of the genus *Orthotospovirus* [26], and its thrips vector *Frankliniella intonsa* (Thysanoptera: Thripidae) to determine how virus infection induces changes in its plant host’s VOCs to attract thrips vectors. TZSV, which causes substantial yield loss in Southwest China, was first isolated in the Yunnan Province in 2008 [27]. TZSV is transmitted by various thrips species: *Frankliniella occidentalis*, *F*. *intonsa*, *F*. *schultzei*, *Thrips palmi*, and *T*. *tabaci* [26]. Flower thrips (*F*. *intonsa*) is the dominant thrips species in several areas of China [28,29] (S1 Fig). Feeding on TZSV-infected plants significantly increased the population size of insect vectors [7]. Furthermore, *F*. *occidentalis* prefers infected plants over mock-inoculated plants, and the relative emissions of volatiles from TZSV-infected plants are significantly higher than those of mock-inoculated plants [30]. Thus, TZSV can induce changes in the emission of plant VOCs that attract thrips vectors, although the nature of the vector olfactory proteins that regulate their behavioural responses to virus-infected plants is unknown.

To elucidate the underlying regulatory mechanism, we performed a series of free-choice experiments to assess *F*. *intonsa* performance on virus-infected plants and the plant volatile cues induced by virus infection. Of four olfactory proteins (FintCSP1, FintCSP2, FintOBP, and FintOR), we demonstrate that FintCSP1 is a key olfactory protein for *F*. *intonsa* to perceive and locate TZSV-infected pepper (*Capsicum annuum*) plants. We also conducted fluorescence ligand binding assays to detect an interaction between specific volatiles and FintCSP1. We carried out homology modeling, molecular docking, site-directed mutagenesis, and ligand binding assays with FintCSP1 to identify the amino acids that contribute to the recognition between the VOC and its receptor. Moreover, CSP is also a key olfactory protein for *F*. *occidentalis* to perceive and locate TZSV-infected pepper plant odors. Our study reveals that a persistent plant virus manipulates the behaviour of its insect vector and this may promote or facilitate onward transmission of the virus. The results presented here provide a means to control the spread of plant viral diseases by modulating insect behaviour.

## Results

### *F*. *intonsa* prefers volatiles emitted by TZSV-infected pepper plants

**Fig 1:**
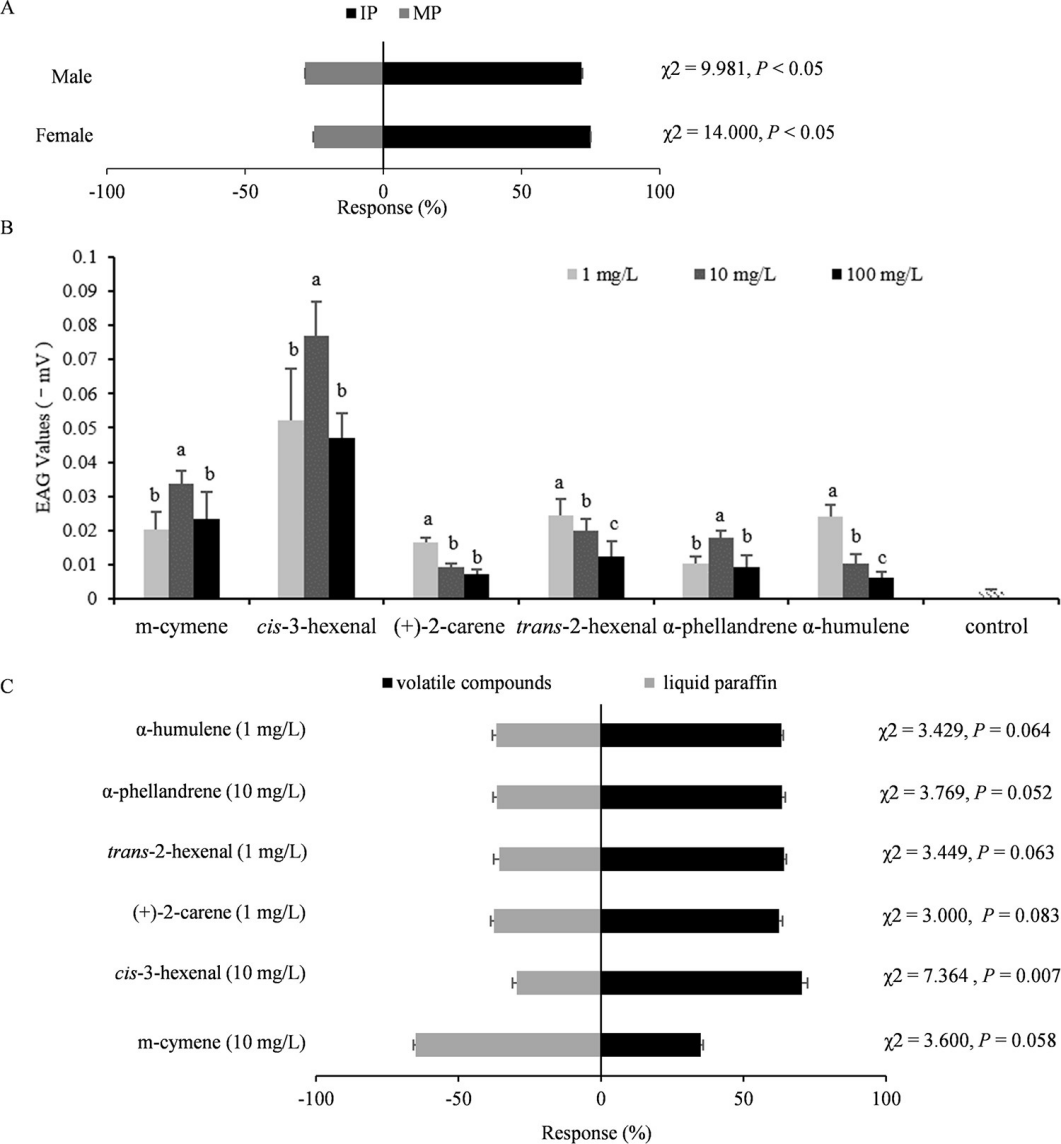
*Frankliniella intonsa* prefers volatiles emitted by TZSV-infected pepper plants. (A) Choice of *F*. *intonsa* on pepper plants that were mock-inoculated (MP) or virus-infected (IP). Significant differences were determined by chi-squared (χ^2^) test (*P* < 0.05). (B) Electroantennogram (EAG) responses of *F*. *intonsa* antennae to volatile compounds. Different lowercase letters indicate significant differences between doses (*P* < 0.05). Ten individuals were assayed per dose and per compound. Data are means ± SE. (C) Preference tests of *F*. *intonsa* to volatile compounds. Data are means ± SE, expressed as a percentage of the number of thrips (*n* = 60) in a Y-tube olfactometer exposed to one of six volatile compounds induced by TZSV infection (right) or liquid paraffin (control, left). Significant differences were determined by χ^2^ tests (*P* < 0.05).

As the dominant thrips species in several areas of China [28,29], *F*. *intonsa* is also an important vector of TZSV (S1 Fig). To examine the response of *F*. *intonsa* to VOC cues from TZSV-infected pepper plants, we assessed the patterns of orientation preference of thrips exposed to plant odors (TZSV-infected or mock-inoculated pepper plants) in the absence of any other visual, taste, or contact cue. We determined that both females (75.0%) and males (71.7%) preferentially choose infected plants, with a significant difference from insects selecting mock-inoculated plants (female, 25.0%, χ^2^ = 14.000, *P*<0.05; male, 28.3%, χ^2^ = 9.981, *P*<0.05; Fig 1A). This result illustrated the preference of *F*. *intonsa* for the odors of TZSV-infected pepper plants.

We previously showed that two volatile compounds, *cis*-3-hexenal and *trans*-2-hexenal, are uniquely emitted by TZSV-infected pepper plants compared to mock-inoculated plants [30]. Furthermore, four volatile compounds, i.e., m-cymene, (+)-2-carene, α-phellandrene, and α-humulene, which are released from TZSV-infected pepper plants, are produced in significantly higher amounts than in mock-inoculated plants [30]. To test whether any of these six volatile compounds might influence or contribute to *F*. *intonsa* preference for TZSV-infected pepper plants, we excised thrips antennae to perform electroantennogram (EAG) analyses. All six volatile compounds triggered an EAG response in our study, although thrips exhibited differences as a function of the odor and its concentration (Fig 1B). The EAG peak amplitudes of *F*. *intonsa* antennae to m-cymene (10 mg/L), *cis*-3-hexenal (10 mg/L), (+)-2-carene (1 mg/L), *trans*-2-hexenal (1 mg/L), α-phellandrene (10 mg/L), and α-humulene (1 mg/L) were approximately –0.034, –0.077, –0.016, –0.024, –0.018, and –0.024 mV, respectively (Fig 1B). In addition, we used Y-tube olfactometers to assess which of these six volatile compounds are attractive to thrips. While all tested odorants showed a tendency to attract *F*. *intonsa* individuals over the liquid paraffin control, only *cis*-3-hexenal reached statistical significance (χ^2^ = 7.364, *P* = 0.007, Fig 1C). Furthermore, m-cymene appeared to repel *F*. *intonsa*, but this result was not statistically significant (χ^2^ = 3.600, *P* = 0.058, Fig 1C). These results demonstrated the preference of *F*. *intonsa* for the specific volatile *cis*-3-hexenal emitted by TZSV-infected pepper plants.

### FintCSP1 is a key olfactory protein involved in modulating the attraction of *F*. *intonsa* to TZSV-infected pepper plant odors

**Fig 2:**
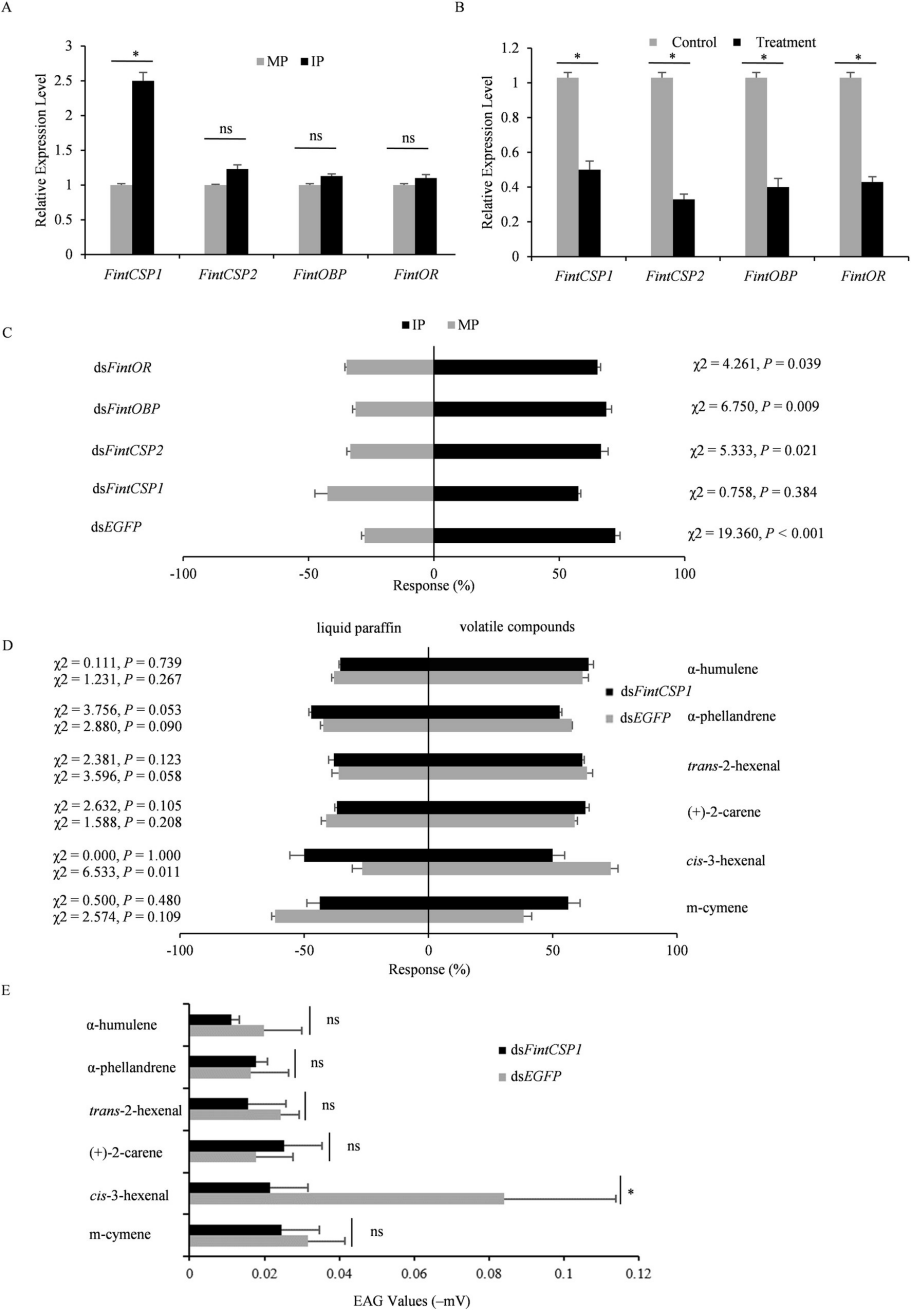
Odorant responses of olfactory genes of *Frankliniella intonsa* after exposure to TZSV-infected pepper plant odors. (A) Relative transcript levels of *FintCSP1*, *FintCSP2*, *FintOBP*, and *FintOR* in *F*. *intonsa* after exposure to TZSV-infected (IP) or mock-inoculated (MP) pepper plants. *, *P* < 0.05, as determined by *t-*test between MP and IP treatments; ns, not significant. Each histogram bar represents the mean (± standard error) of three replicates. (B) Relative transcript levels of *FintCSP1*, *FintCSP2*, *FintOBP*, and *FintOR* in *F*. *intonsa* injected with ds*FintCSP1*, ds*FintCSP2*, ds*FintOBP*, ds*FintOR*, and ds*EGFP* (control). Each gene contained three replicates. *, *P* < 0.05, as determined by *t-*test between control and treatment. Each histogram bar represents the mean (± standard error) of three replicates. (C) Behavioural responses of *F*. *intonsa* to plants after injection with ds*FintCSP1*, ds*FintCSP2*, ds*FintOBP*, ds*FintOR*, and ds*EGFP* (control). Significant differences were determined by chi-squared (χ^2^) test (*P* < 0.05). (D) Behavioural responses of thrips to volatile compounds induced by TZSV infection (right) or liquid paraffin (control, left) after injection with the indicated dsRNA. Significant differences between the treatment (ds*FintCSP1*) and the control (ds*EGFP*) were determined by χ^2^ test (*P* < 0.05). (E) EAG responses of dsRNA-injected *F*. *intonsa* to individual volatile compounds. *, *P* < 0.05, as determined by *t-*test between the treatment (ds*FintCSP1*) and the control (ds*EGFP*); ns, not significant.

To identify the key olfactory genes in *F*. *intonsa* that help the insects locate TZSV-infected pepper plants, we characterized their relative expression levels after exposure to TZSV-infected or mock-inoculated pepper plant odors. Based on GenBank data deposited at the National Center for Biotechnological Information (NCBI) database, we selected four olfactory genes, *FintCSP1*, *FintCSP2*, *FintOBP*, and *FintOR*. After exposing *F*. *intonsa* individuals to TZSV-infected plant odors for 12 h, *FintCSP1* expression increased compared to insects exposed to mock-inoculated plant odors (*P*<0.05), while the other three genes did not reach statistical significance (Fig 2A). To further explore which *F*. *intonsa* olfactory genes participate in the host preference, we silenced each gene via RNA interference (RNAi) by injecting double-stranded RNAs (dsRNAs) (Fig 2B). In the Y-tube olfactometer assay, thrips with lower transcript levels for *FintCSP2*, *FintOBP*, or *FintOR* still exhibited a preference for TZSV-infected plants, as did insects injected with a dsRNA targeting the enhanced green fluorescent protein gene (*EGFP*) (*P*<0.05, Fig 2C). By contrast, ds*FintCSP1*-injected *F*. *intonsa* displayed no clear preference for either plant (χ^2^ = 0.758, *P* = 0.384, Fig 2C). These results indicated that FintCSP1 functions in the olfactory response of *F*. *intonsa* to TZSV-infected pepper plants.

To clarify the function of FintCSP1 in *F*. *intonsa* in TZSV-infected pepper plant odor perception, we employed the olfactometer and EAG assays to evaluate the responses to the six volatile compounds mentioned earlier. Silencing of *FintCSP1* only resulted in behavioural changes in *F*. *intonsa* in response to *cis*-3-hexenal in the olfactometer assay (Fig 2D). In ds*EGFP*-treated thrips, 73.3% of individuals recognized *cis*-3-hexenal, with the remaining 26.7% moving toward the liquid paraffin control (χ^2^ = 6.533, *P* = 0.011, Fig 2D). *FintCSP1-*silenced thrips showed no preference between the liquid paraff in control and *cis*-3-hexenal (χ^2^ = 0.000, *P* = 1.000, Fig 2D). To validate the altered behaviour observed for *FintCSP1-*silenced *F*. *intonsa* in the olfactometer assay, we excised thrips antennae and exposed them to each volatile compound. Compared to injection with ds*EGFP*, *FintCSP1-*silenced antennae showed a significantly lower response to *cis*-3-hexenal (*F* = 2.777; *df* = 16, *P* = 0.028, Fig 2E). Moreover, *cis*-3-hexenal was the only compound to which the antennae of ds*FintCSP1-*injected *F*. *intonsa* showed a different response relative to ds*EGFP*-injected thrips (Fig 2E). These observations indicated that FintCSP1 is a key olfactory protein of *F*. *intonsa* to modulate the thrips’ response to *cis*-3-hexenal.

### FintCSP1 is abundant in antennae of *F*. *intonsa*

To investigate how FintCSP1 detects and transports volatile compounds emitted by TZSV-infected plants, we cloned and sequenced the full-length *FintCSP1* coding sequence (S2 Fig). The predicted open reading frame was 405 bp, encoding a protein of 135 amino acids, including a predicted signal peptide of 19 amino acids at the N-terminus (S3 Fig). The predicted isoelectric point (pI) and molecular weight of FintCSP1 were 8.13 and 15.01 kDa, respectively. Moreover, FintCSP1 contained four conserved cysteines (S3 Fig), which was consistent with the typical conserved characteristics of CSPs: C1-X8-C2-X18-C3-X2-C4 (with X representing any amino acid except cysteine) [31–33]. Subsequently, we selected 31 CSPs from eight insect orders to construct a phylogenetic tree using the neighbor-joining method with Mega 7.0: Orthoptera (eleven sequences), Hemiptera (one sequence), Lepidoptera (one sequence), Thysanoptera (two sequences), Coleoptera (eight sequences), Hymenoptera (three sequences), Blattaria (two sequences), and Diptera (three sequences) (S4 Fig). This phylogenetic analysis showed that FintCSP1 clusters within a small branch close to CSP from *F*. *occidentalis*.

**Fig 3:**
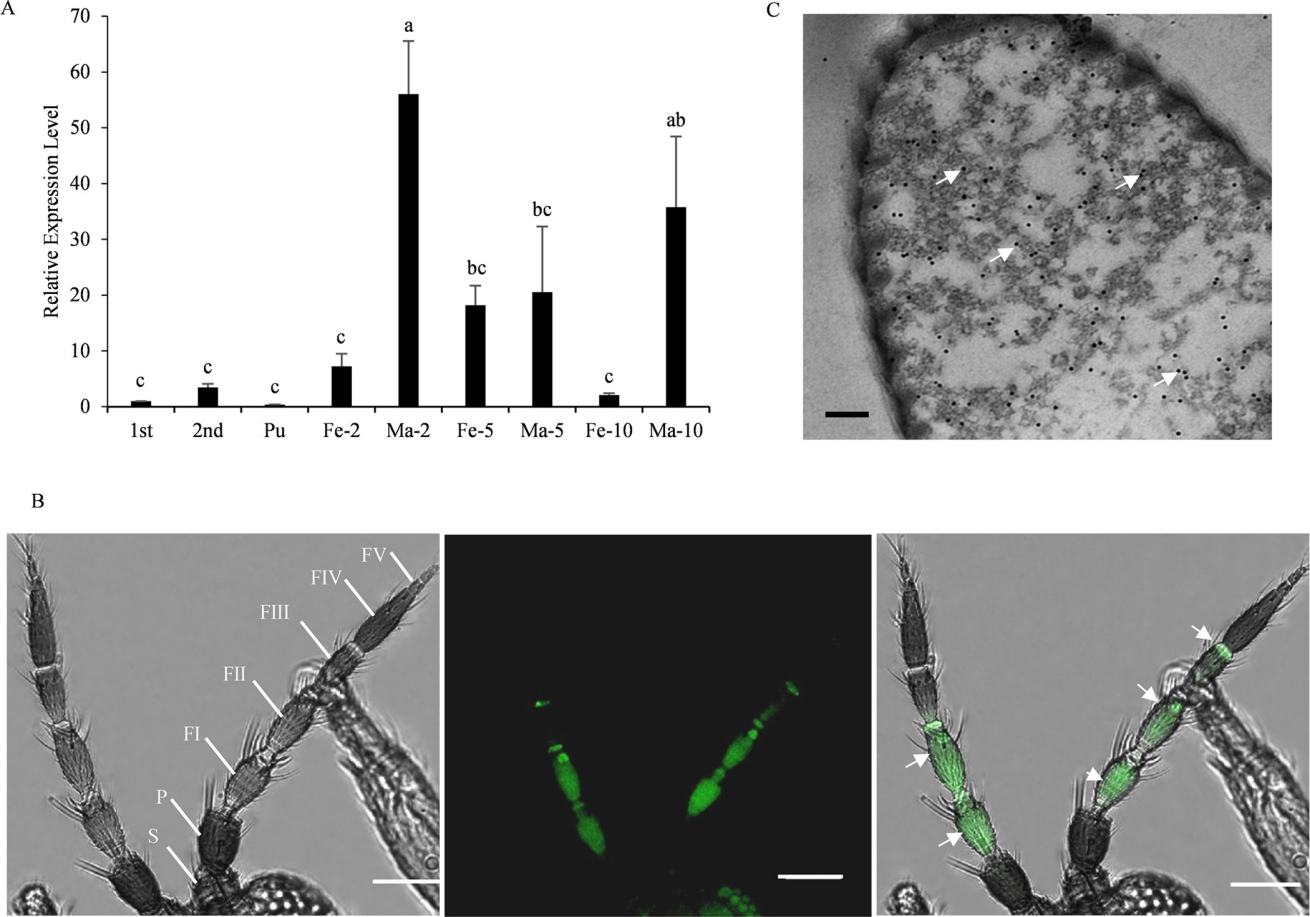
*FintCSP1* and FintCSP1 abundance in antennae of *Frankliniella intonsa*. (A) *FinCSP1* relative transcript levels in antennae at various developmental stages of *F*. *intonsa*. Data are means ± SE. Different lowercase letters indicate significant differences, as determined by one-way ANOVA (*P* < 0.05). 1st: 1 instar nymphs; 2nd: 2 instar nymphs; Pu: pupae; Fe-2, Fe-5, Fe-10: 2-, 5-, and 10-day-old female adults; Ma-2, Ma-5, Ma-10: 2-, 5-, and 10-day-old male adults. (B) Distribution of FintCSP1 in female antennae of *F*. *intonsa*. Thrips were immunostained with FintCSP1-FITC (green) and observed under a confocal laser scanning microscope. FI-V: Flagellomeres I-V; P: pedicel; S: scape. Scale bars, 50 μm. (C) Immunolocalization of FintCSP1 in female adult antennae of *F*. *intonsa*. Sensilla basiconica in flagellum I was immunolabeled with FintCSP1-specific IgG as the primary antibody, followed by a specific secondary antibody that had been conjugated with 12-nm gold particles (white arrows). Scale bar, 200 nm.

We determined *FintCSP1* transcript levels in antennae at various developmental stages of *F*. *intonsa* by reverse transcription quantitative PCR (RT-qPCR), setting the levels in 1-day-old first-instar nymphs to 1 for normalization. *FintCSP1* was expressed in all developmental stages examined, with the highest expression in the 2-day-old male adults, which was 56.05-fold higher than that in 1-day-old first-instar nymphs (Fig 3A). FintCSP1 also accumulated in female adult antennae, as revealed by immunofluorescence microscopy and immunoelectron microscopy experiments (Fig 3B and 3C). The antennae of female adult *F*. *intonsa* contain scape, pedicel, and a long flagellum with five flagellomeres (I–V) (Fig 3B). We detected strongly fluorescent foci at the three flagellomeres (I–III) of antenna, while no fluorescent signal was observed in the other parts (Fig 3B). In addition, using immunogold labeling with FintCSP1-specific rabbit polyclonal antibody, we detected FintCSP1 proteins in sensilla basiconica at the flagellum I (Fig 3C), indicating that FintCSP1 is abundant in antennae.

### FintCSP1 displays a moderate binding affinity to *cis*-3-hexenal

**Fig 4:**
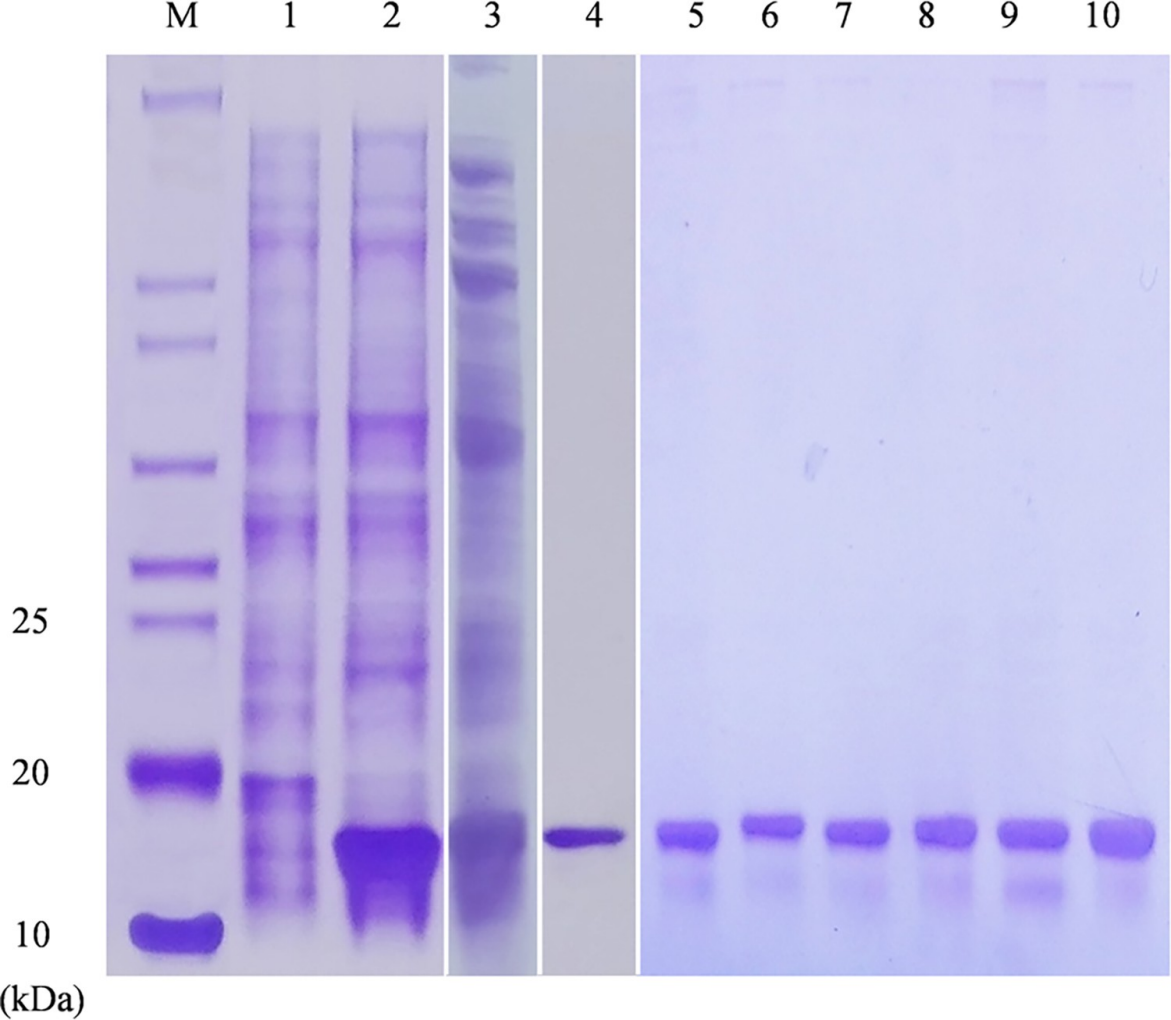
SDS-PAGE analysis of recombinant target proteins. M, Molecular weight marker; 1, non-induced pET30a/FintCSP1; 2, induced crude extract from pET30a/FintCSP1; 3, supernatant of pET30a/FintCSP1; 4, purified recombinant wild-type FintCSP1 protein; 5−10, purified recombinant variants harboring the individual mutations Lys26Ala, Phe27Ala, Thr28Ala, Glu67Ala, Ser84Ala, and Val132Ala, respectively.

To characterize the biochemical binding activity of FintCSP1 to volatile substances, we produced a purified recombinant FintCSP1 (Fig 4). We detected a single band by sodium dodecyl sulfate polyacrylamide gel electrophoresis (SDS-PAGE) with an apparent molecular weight between 10 and 20 kDa, which was consistent with its predicted molecular weight of 15.01 kDa (Fig 4).

**Fig 5:**
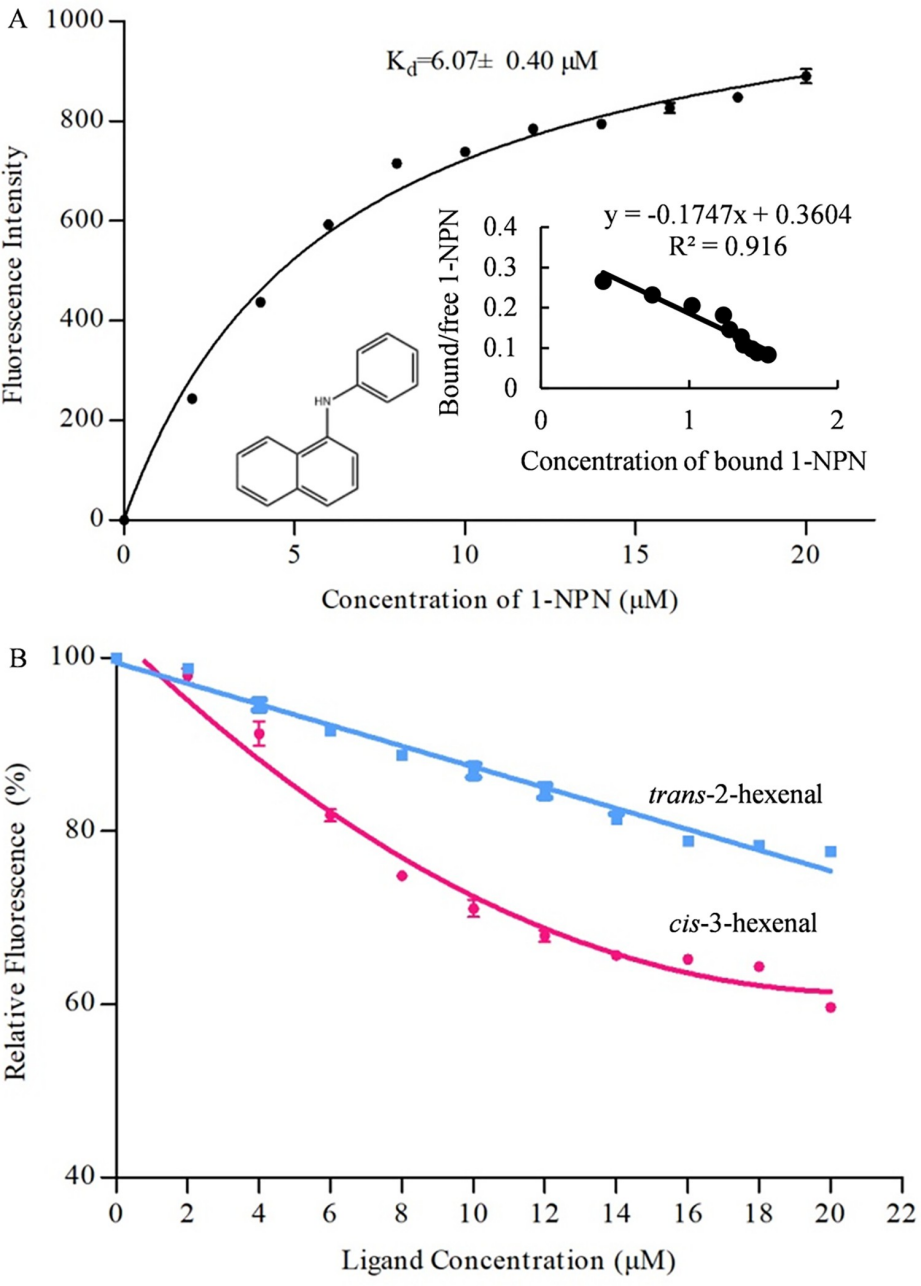
Binding of 1-NPN to FintCSP1 and competitive binding of FintCSP1 with ligands. (A) Binding curve of 1-NPN to FintCSP1. Inset: Scatchard plot analysis. (B) Binding assays between recombinant FintCSP1 and *cis*-3-hexenal or *trans*-2-hexenal. A mixture of recombinant FintCSP1 protein and 1-NPN in 50 mM Tris-HCl buffer (pH 7.4) both at a concentration of 2 μM was titrated with each competing ligand over a final concentration range of 2–20 μM. Data represent the means of three independent replicates. Error bars indicate SE.

N-phenyl-1-naphthylamine (1-NPN) is frequently used as a fluorescent probe to assess the affinity and specificity of olfactory proteins for potential ligands [19]. Binding plots for FintCSP1 indicated that the binding of 1-NPN is saturable, with a binding constant (K_d_) of 6.07 ± 0.40 μM at pH 7.4 (Fig 5A). Therefore, 1-NPN would provide an adequate fluorescent probe in competitive binding assays of purified FintCSP1 to different ligands. We thus used a competitive fluorescence binding assay to determine the binding affinity of FintCSP1 to *cis*-3-hexenal and its stereo-isomer *trans*-2-hexenal (Fig 5B). We calculated the median inhibitory concentration (IC_50_) and dissociation constant (K_i_) values, which revealed that FintCSP1 displays a moderate binding affinity to *cis*-3-hexenal (K_i_ = 28.68 ± 0.79 μM), while we observed no FintCSP1 binding to *trans*-2-hexenal, for which K_i_ values were >100 μM (Fig 5B and S2 Table). Together, our results indicated that FintCSP1 binds *cis*-3-hexenal, which attracts *F*. *intonsa*.

### *Cis*-3-hexenal tightly binds to residues of the FintCSP1

**Fig 6:**
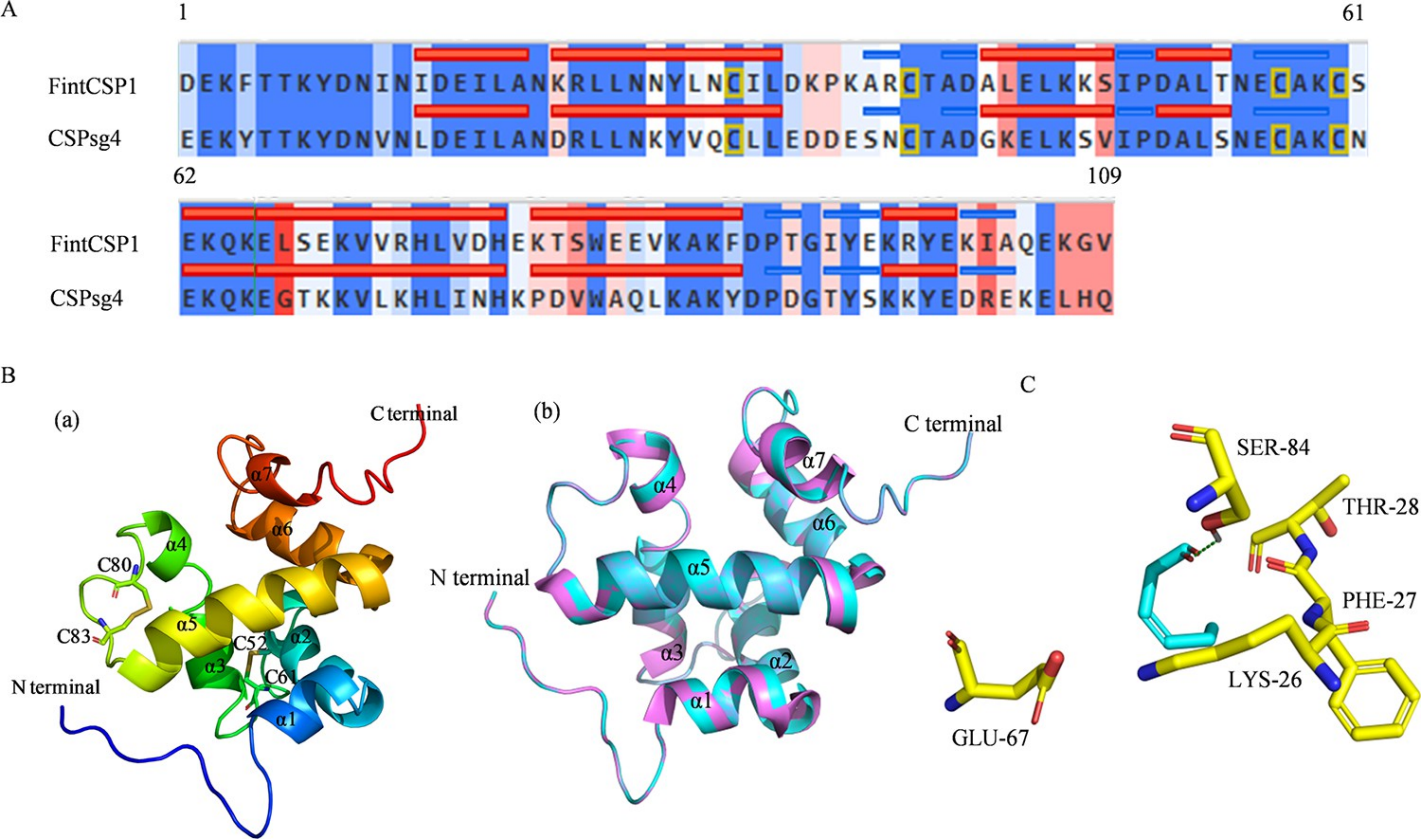
Homology modeling and molecular docking analysis for FintCSP1. (A) Structure-based sequence alignment between FintCSP1 and the template (CSPsg4) structure. Identical or similar residues are highlighted in blue, and dissimilar ones are highlighted in red; darker colors indicate more similar or dissimilar residues. Residues corresponding to α-helix regions are marked by horizontal red lines; random coil or turn regions are marked by horizontal blue lines. Conserved cysteines are marked by orange box. (B) Predicted 3D model of FintCSP1 (a) and superposed FintCSP1 model onto the template structure (b). FintCSP1 is shown in cyan, and the template structure is shown in violet. (C) Interaction diagram between FintCSP1 amino acid residues and *cis*-3-hexenal. *Cis*-3-hexenal is shown in cyan. Surrounding residues in the binding pocket are colored in yellow. Hydrogen bond is depicted as a green dashed line.

To identify which amino acids play an important role in the interaction between FintCSP1 and *cis*-3-hexenal, we carried out homology modeling and molecular docking analyses for FintCSP1. As the chemosensory protein sg4 from the desert locust (*Schistocerca gregaria*) (CSPsg4; PDB ID: 2GVS) shared 56.9% sequence identity with FintCSP1, we used its three-dimensional (3D) structure as a template to model FintCSP1 (Fig 6A and 6B-a). The Ramachandran plot for FintCSP1 showed that 94.5% of all residues resided in the core region, 5.5% of all residues were within the additionally allowed region, with 100% of all residues in the generously favored region, and no residues in the disallowed region, indicating that the 3D structure of the model is reasonable (S5A Fig). The simulated 3D structure of FintCSP1 consisted of seven α-helixes: α1 (residues Ile36–Ala41), α2 (residues Lys43–Leu54), α3 (residues Ala65–Ser71), α4 (residues Asp74–Thr77), α5 (residues Glu85–His101), α6 (residues Lys103–Phe113), and α7 (residues Lys121–Glu124); and two disulfide bridges: Cys52-Cys61 and Cys80-Cys83 (Fig 6A). The root-mean-square deviation (RMSD) from the superposition between FintCSP1 and the template structure was 0.000Å (109 residues), indicating that the modeling of FintCSP1 is very similar to that of CSPsg4 (Fig 6B-b).

To understand how FintCSP1 interacts with host compounds, we conducted molecular docking predictions to identify the key sites in FintCSP1 that bind to *cis*-3-hexenal. The binding energy between FintCSP1 and *cis*-3-hexenal was –4.2759 kcal/mol (S3 Table). *Cis*-3-hexenal laid deep inside the binding pocket of FintCSP1, exhibiting bonded interactions (Fig 6C). Within the binding pocket, the oxygen atom of *cis*-3-hexenal, the possible hydrogen bond receptor, formed a hydrogen bond with the oxygen atom in the side chain of Ser84 in FintCSP1 (S3 Table). In addition, Van Der Waals interactions also formed between *cis*-3-hexenal and four residues, Lys26, Phe27, Thr28, and Glu67 (S3 Table). These interactions mainly contributed to the binding energy between *cis*-3-hexenal and FintCSP1, indicating that *cis*-3-hexenal tightly binds in the center of the FintCSP1 pocket and influences FintCSP1 activity.

### Three amino acids in FintCSP1 affect its binding to *cis*-3-hexenal

**Fig 7:**
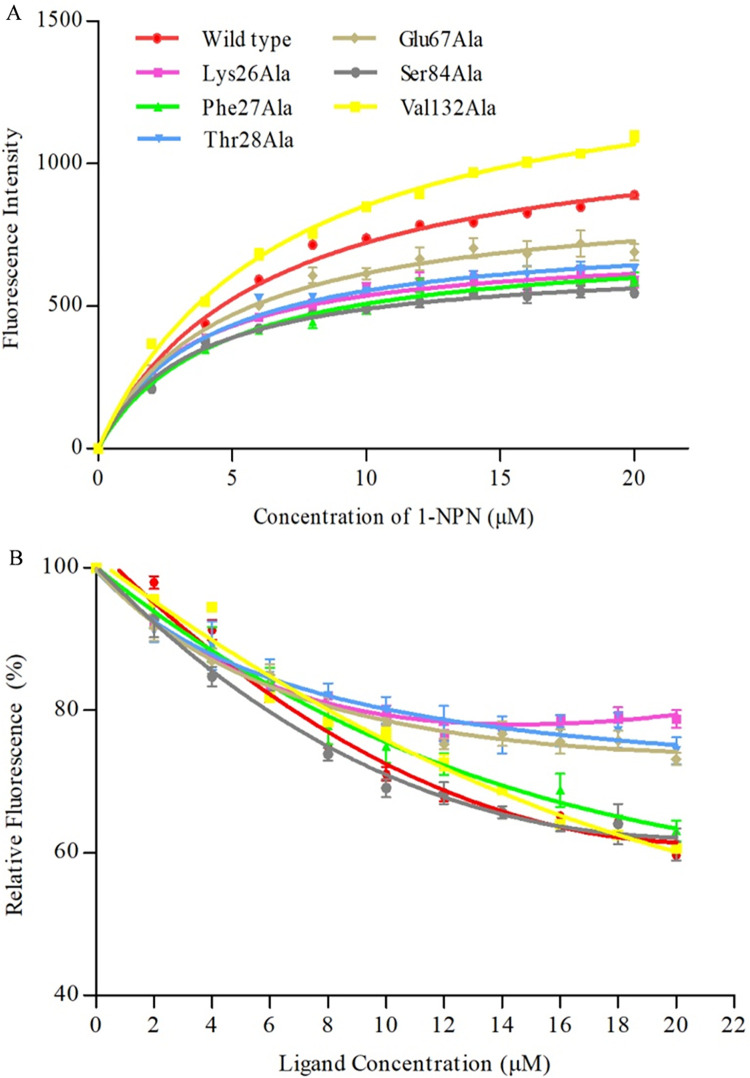
Binding curves of wild-type FintCSP1 and selected mutants to *cis*-3-hexenal. (A) Binding curve of 1-NPN to wild-type FintCSP1 or each of the six variants. (B) Competitive binding curves between wild-type FintCSP1 or variants and *cis*-3-hexenal. A mixture of recombinant wild-type FintCSP1 and mutant protein and 1-NPN in 50 mM Tris-HCl buffer (pH 7.4) both at 2 μM was titrated with each competing ligand over the concentration range of 2–20 μM. Data represent means of three independent replicates. Error bars indicate SE.

To validate the molecular docking results and clarify the roles of the five predicted key amino acid sites of FintCSP1 in binding to *cis*-3-hexenal, we produced recombinant FintCSP1 proteins harboring single amino acid substitutions to alanine (Ala) at each of the five key residues (Fig 4). We performed fluorescent binding assays with 1-NPN and wild-type FintCSP1 or its variants. The binding constants (K_d_) of mutant proteins with 1-NPN were 3.30 ± 0.39 μM for Lys26Ala, 4.38 ± 0.49 μM for Phe27Ala, 3.85 ± 0.41 μM for Thr28Ala, 4.50 ± 0.61 μM for Glu67Ala, 3.48 ± 0.33 μM for Ser84Ala, and 6.77 ± 0.36 μM for Val132Ala, indicating that all variants bind to 1-NPN well, reaching 54% to 111.5% of the binding seen with intact FintCSP1 (Fig 7A). We conducted a competitive fluorescent assay between 1-NPN, *cis*-3-hexenal, and each of the FintCSP1 variants. We discovered that FintCSP1-Ser84Ala and FintCSP1-Val132Ala exhibit a similar binding ability to *cis*-3-hexenal as wild-type FintCSP1, whereas FintCSP1-Lys26Ala, FintCSP1-Thr28Ala, and FintCSP1-Glu67Ala had no affinity for *cis*-3-hexenal (Fig 7B and Table 1). In addition, FintCSP1-Phe27Ala showed a weaker affinity for *cis*-3-hexenal than wild-type FintCSP1 (Fig 7B and Table 1). These results indicated that three amino acids in FintCSP1 (Lys26, Thr28, and Glu67) affect its binding to *cis*-3-hexenal.

**Table 1:** Binding affinities of *cis*-3-hexenal to wild-type FintCSP1 and mutants.

Protein	IC_50_ (μM)	K_i_ (μM)
Wild type	36.15 ± 1.0	28.68 ± 0.79b
Lys26Ala	nd	nd
Phe27Ala	53.47 ± 3.07	41.13 ± 2.36a
Thr28Ala	nd	nd
Glu67Ala	nd	nd
Ser84Ala	41.28 ± 3.96	29.65 ± 2.84b
Val132Ala	39.24 ± 0.36	32.18 ± 0.29b

Affinities are given as means ± standard error (SE). IC_50_, ligand concentration displacing 50% of the fluorescence intensity of the FintCSP1/1-NPN complex; K_i_, dissociation constant; nd, not determined (binding constant was not calculated; IC_50_ > 100 μM). Data within a column followed by different lowercase letters are significantly different (Tukey’s HSD test; *P* < 0.05).

### CSP is also a key olfactory protein for *F*. *occidentalis* to perceive and locat TZSV-infected pepper plants

**Fig 8:**
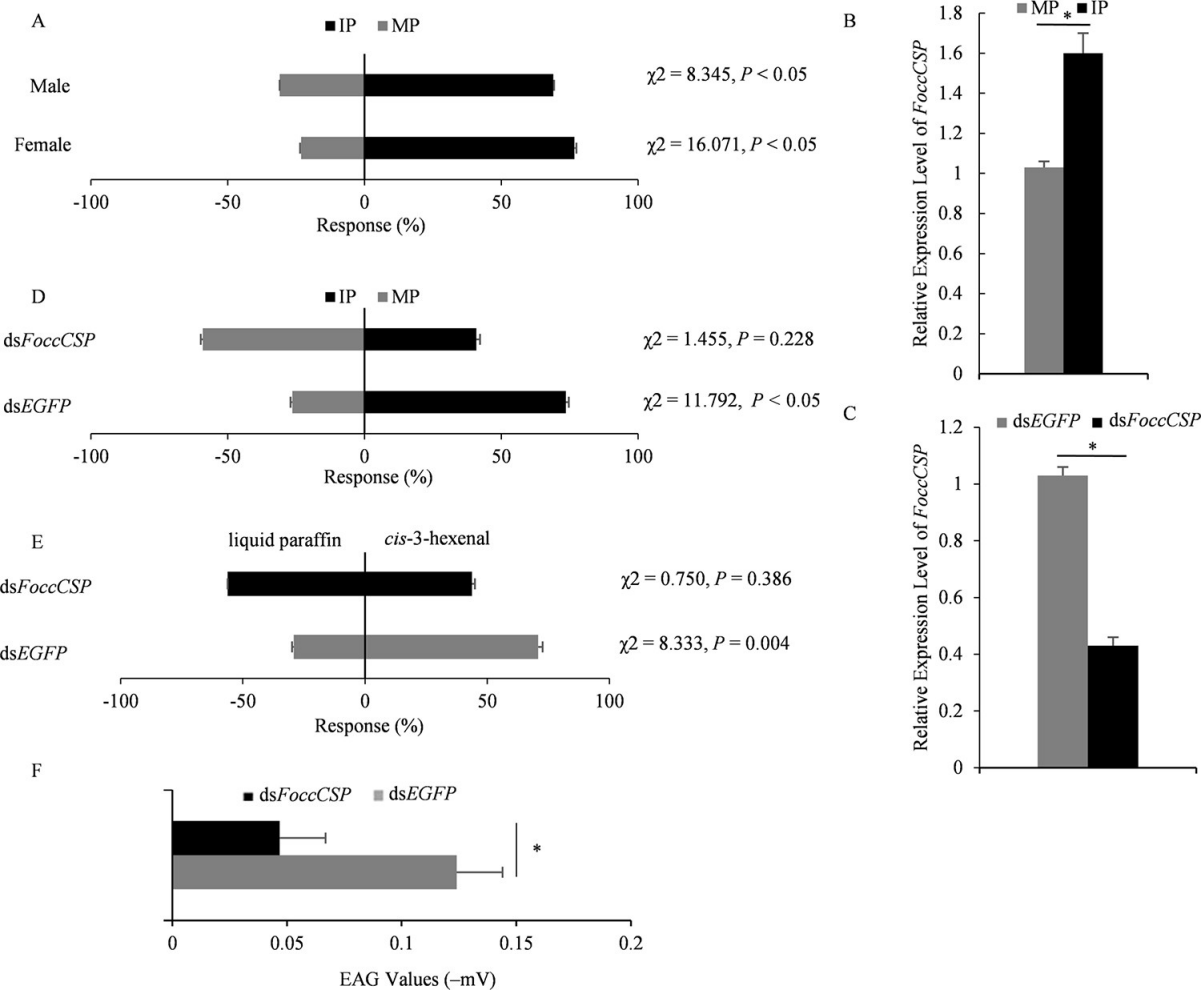
*Frankliniella occidentalis* prefers volatile cues from TZSV-infected pepper plants. (A) Choice of *F*. *occidentalis* on pepper plants that were mock-inoculated (MP) or virus–infected (IP). Significant differences were determined by chi-squared (χ^2^) test (*P* < 0.05). (B) Relative transcript levels of *FoccCSP* in *F*. *occidentalis* after exposure to TZSV-infected (IP) or mock-inoculated (MP) pepper plants. *, *P* < 0.05, as determined by *t-*test between MP and IP treatments. Each histogram bar represents the mean (± standard error) of three replicates. (C) Relative transcript levels of *FoccCSP* in *F*. *occidentalis* injected with ds*FoccCSP* and ds*EGFP* (control). Each histogram bar represents the mean (± standard error) of three replicates. *, *P* < 0.05, as determined by *t-*test between control and treatment. (D) Behavioural responses of *F*. *occidentalis* to mock-inoculated (MP) or virus-infected (IP) plants after injection with ds*FoccCSP* or ds*EGFP* (control). Significant differences were determined by χ^2^ test (*P*< 0.05). (E) Behavioural responses of thrips to *cis*-3-hexenal (right) or liquid paraffin (control, left) after injection with ds*FoccCSP* or ds*EGFP*. Significant differences between the treatment (ds*FoccCSP*) and the control (ds*EGFP*) were determined by χ^2^ test (*P* < 0.05). (F) EAG responses of ds*FoccCSP*-injected *F*. *occidentalis* to *cis*-3-hexenal. *, *P* <0.05, as determined by *t-*test between the treatment (ds*FoccCSP*) and the control (ds*EGFP*).

To validate the response of *F*. *occidentalis* to VOC cues from TZSV-infected pepper plants, we assessed the patterns of orientation preference of thrips exposed to plant odors (TZSV-infected or mock-inoculated pepper plants) in the absence of any other visual, taste, or contact cue. Both females (76.8%) and males (69.0%) preferentially chose infected plants, and the result was significantly different from that of insects selecting mock-inoculated plants (female, 23.2%, χ^2^ = 16.071, *P* < 0.05; male, 31.0%, χ^2^ = 8.345, *P* < 0.05; Fig 8A). This result illustrated the preference of *F*. *occidentalis* for the odors of TZSV-infected pepper plants.

After exposing *F*. *occidentalis* individuals to TZSV-infected plant odors for 12 h, the expression levels of *FoccCSP* increased compared to insects exposed to mock-inoculated plant odors (*P* < 0.05, Fig 8B). Then, we silenced *FoccCSP* in *F*. *occidentalis* via RNAi as done in *F*. *intonsa* (Fig 8C). In the Y-tube olfactometer assay, *FoccCSP*-silenced *F*. *occidentalis* displayed no clear preference for either plant (Fig 8D). In addition, ds*FoccCSP*-silenced *F*. *occidentalis* displayed no clear preference for *cis*-3-hexenal or the liquid paraffin control (Fig 8E). Furthermore, compared to injection with ds*EGFP*, *FoccCSP*-silenced antennae showed a significantly lower response to *cis*-3-hexenal (*F* = 0.306; *df* = 5, *P* = 0.034, Fig 8F). These results indicated that FoccCSP functions in the olfactory response of *F*. *occidentalis* to TZSV-infected pepper plants.

To characterize the biochemical binding activity of FoccCSP to *cis*-3-hexenal, we produced a purified recombinant FoccCSP protein (S6 Fig). Binding plots for FoccCSP indicated that the binding of 1-NPN is saturable, with a K_d_ of 6.36 ± 0.51 μM at pH 7.4 (S7 Fig). Recombinant FoccCSP specifically bound to *cis*-3-hexenal, with a K_i_ of 23.42 ± 0.36 μM (Table 2). Furthermore, compared to other crystal structures of insect CSPs, *S*. *gregaria* CSPsg4 shared higher amino acid identity with FoccCSP (S8A Fig). The Ramachandran plot for FoccCSP indicated that nearly all the residues were located at the rational region (S5B Fig). Additionally, the simulated FoccCSP 3D structure consisted of seven α-helixes, and two disulfide bridges with the exact same residues as those of FintCSP1 with the exception of α5 residues (Glu85-Lys101) (S8A and S8B Fig).

**Table 2:** Binding affinities of *cis*-3-hexenal to wild-type FoccCSP and mutants.

Protein	IC_50_ (μM)	K_i_ (μM)
Wild type	28.77 ± 0.45	23.42 ± 0.36e
Lys26Ala	35.96 ± 0.79	32.64 ± 0.71d
Phe27Ala	nd	nd
Thr28Ala	nd	nd
Thr29Ala	nd	nd
Tyr31Ala	43.91 ± 0.66	37.44 ± 0.56c
Asp64Ala	65.93 ± 0.79	62.21 ± 0.75b
Glu67Ala	92.89 ± 2.14	83.65 ± 1.93a
Gln87Ala	47.47 ± 0.44	43.68 ± 0.41c
Val132Ala	28.70 ± 0.12	23.69 ± 0.10e

Affinities are given as means ± standard error (SE). IC_50_, ligand concentration displacing 50% of the fluorescence intensity of the FoccCSP/1-NPN complex; K_i_, dissociation constant; nd, not determined (binding constant was not calculated; IC_50_ > 100 μM). Data within a column followed by different lowercase letters are significantly different (Tukey’s HSD test; *P* < 0.05).

**Fig 9:**
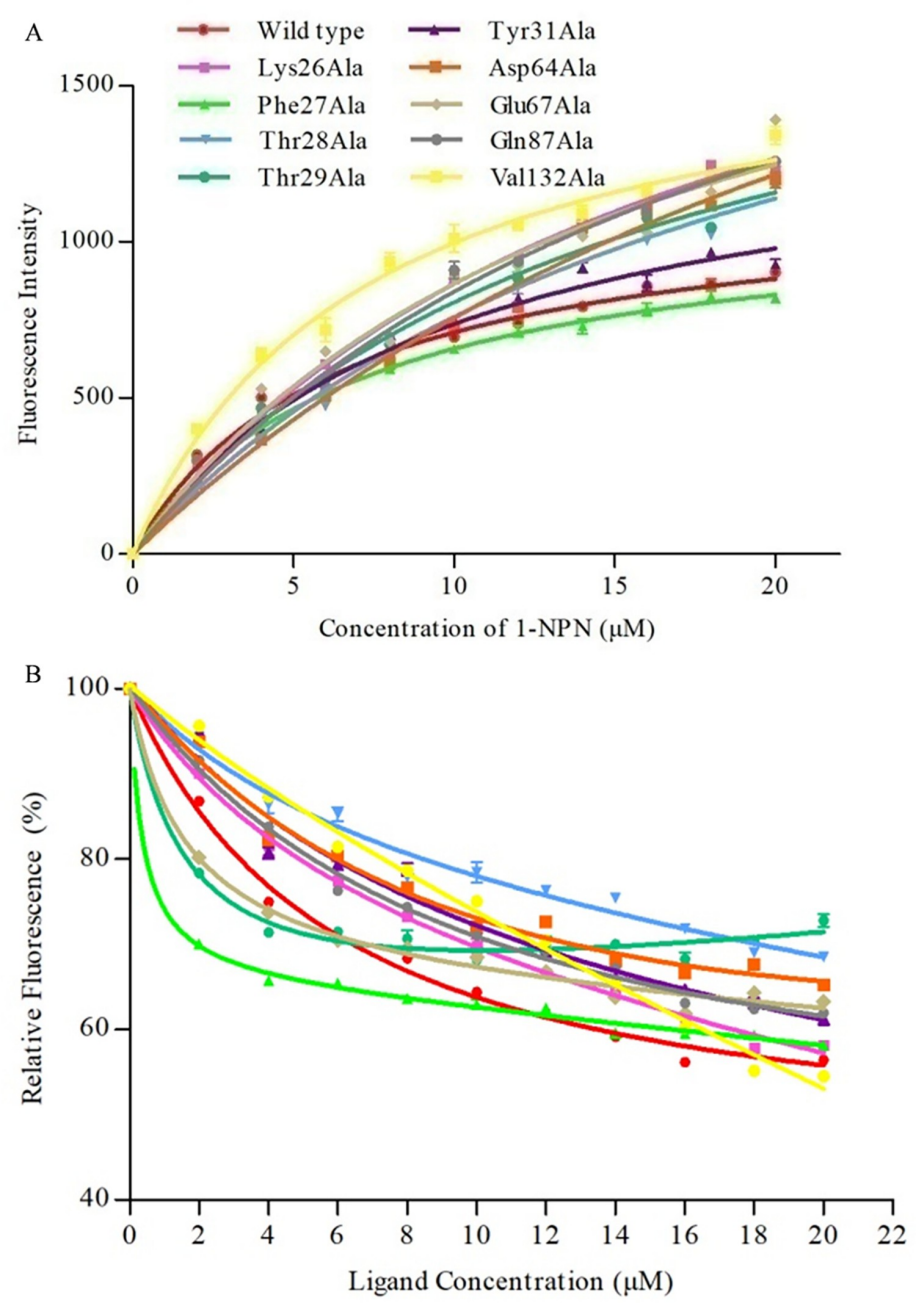
Binding curves of wild-type FoccCSP and selected mutants to *cis*-3-hexenal. (A) Binding curve of 1-NPN to wild-type FoccCSP or each of the nine variants. (B) Competitive binding curves between wild-type FoccCSP or variants and *cis*-3-hexenal. A mixture of recombinant wild-type FoccCSP and mutant protein and 1-NPN in 50 mM Tris-HCl buffer (pH 7.4) both at 2 μM was titrated with each competing ligand over the concentration range of 2–20 μM. Data represent means of three independent replicates. Error bars indicate SE.

The molecular docking results showed that the binding energy between FoccCSP and *cis*-3-hexenal was –4.0882kcal/mol (S4 Table). *Cis*-3-hexenal formed a suitable steric complementarity with the binding site of FoccCSP, exhibiting bonded interactions (S8C Fig). Within the binding pocket, the oxygen atom of *cis*-3-hexenal, the possible hydrogen bond receptor, formed a hydrogen bond with the nitrogen atom in the backbone of Phe27 in FoccCSP (S4 Table). In addition, Van Der Waals interactions also formed between *cis*-3-hexenal and seven residues, Lys26, Thr28, Thr29, Tyr31, Asp64, Glu67, and Gln87 (S4 Table). Moreover, to verify the results of molecular docking and clarify the key amino acids associated with binding activities of FoccCSP to *cis*-3-hexenal, we performed a site-directed mutation assay and prepared the purified FoccCSP mutant proteins (S6 Fig). The K_d_ of mutant proteins with 1-NPN were 17.36 ± 1.32 μM for Lys26Ala, 7.09 ± 0.39 μM for Phe27Ala, 19.89 ± 2.43 μM for Thr28Ala, 15.47 ± 1.74 μM for Thr29Ala, 9.79 ± 0.87 μM for Tyr31Ala, 31.04 ± 4.55 μM for Asp64Ala, 15.73 ± 2.30 μM for Glu67Ala, 20.29 ± 2.25 μM for Gln87Ala, and 7.26 ± 0.66 μM for Val132Ala (Fig 9A). Compared to the wild-type protein, three mutants, FoccCSP-Phe27Ala, FoccCSP-Thr28Ala, and FoccCSP-Thr29Ala, showed no affinity for *cis*-3-hexenal, while the FoccCSP-Val132Ala mutant had only a minor change in binding to *cis*-3-hexenal (Fig 9B and Table 2). In addition, five mutants, FoccCSP-Lys26Ala, FoccCSP-Tyr31Ala, FoccCSP-Asp64Ala, FoccCSP-Glu67Ala, and FoccCSP-Gln87Ala, showed significantly decreased binding affinities to *cis*-3-hexenal (Fig 9B and Table 2). These results indicated that eight amino acids in FoccCSP (Lys26, Phe27, Thr28, Thr29, Tyr31, Asp64, Glu67, and Gln87) affect its binding to *cis*-3-hexenal. Our results revealed a common mechanism by which TZSV induces the production of high amounts of specific plant volatiles that are recognized by thrips CSPs, increasing the attractiveness of infected plants to thrips vectors.

## Discussion

Arboviruses rely on arthropod vectors for transmission among plant hosts [1,3]. To maximize their transmission rates, some plant viruses induce changes in the composition and volume of plant VOCs to make infected plants more attractive to insect vectors, which may promote disease transmission [2,5,12,14]. *Orthotospoviruses*, such as TZSV and tomato spotted wilt virus (TSWV), manipulate the volatiles produced by their host such that infected plants are more attractive to vectors; however, infection by these viruses also negatively affects plant size and fruit quality [5,30]. However, the underlying mechanism of how these viruses manipulate host volatiles has not been elucidated. Here, we demonstrated that, in the absence of any visual, taste, or contact cues, infection with TZSV made pepper plants more attractive the thrips species *F*. *intonsa* and *F*. *occidentalis*, two important TZSV vectors, indicating that these insects possess an innate preference for the blend of volatile compounds emitted by TZSV-infected pepper plants (Figs 1A and 8A). We further showed that of the six volatile compounds produced by TZSV-infected pepper plants in greater amounts compared to mock-inoculated plants, only *cis*-3-hexenal attracted *F*. *intonsa* (Fig 1C). *Cis*-3-hexenal is usually produced in response to mechanical wounding or herbivory and attracts *Anagrus nilaparvatae*, an egg parasitoid of rice planthoppers (*Nilaparvata lugens*), at the very low concentration of 1:20,000 dilution [34,35]. In addition to raising the sensitivity off all webworm moth (*Hyphantria cunea*) antennae to pheromones [36], we showed here that *cis*-3-hexenal triggers an EAG response from *F*. *intonsa* antennae (Figs 1B and 2E), underscoring the involvement of the insect olfactory system in identifying this unique compound. In agreement, we detected abundant FintCSP1 in *F*. *intonsa* antennae, and exposure to TZSV-infected plant odors induced *FintCSP1* and *FoccCSP* expression (Figs 2A, 3 and 8B). To silencing *FintCSP1* or *FoccCSP* transcript levels via RNAi abolished the preference for TZSV-infected plants, suggesting their loss of VOC perception (Figs 2C and 8D). Furthermore, *FintCSP1* or *FoccCSP* silencing reduced the attraction of *F*. *intonsa* or *F*. *occidentalis* to *cis*-3-hexenal and diminished the EAG response of *F*. *intonsa* or *F*. *occidentalis* antennae to *cis*-3-hexenal (Figs 2D, 2E, 8E, and 8F). We thus concluded that FintCSP1 and FoccCSP have crucial roles in the olfactory response of *F*. *intonsa* and *F*. *occidentalis* to *cis*-3-hexenal, thereby helping *F*. *intonsa* and *F*. *occidentalis* identify TZSV-infected plants, which would promote the spread of this persistently transmitted virus.

Chemosensory proteins play critical roles in the insect olfactory system, and are responsible for capturing and transporting outside odorants through hydrophilic lymph to olfactory receptors [37]. In our study, *FintCSP1* was highly expressed in female adults and *FintCSP1* mRNA levels increased rapidly up to day 5 after emergence and then began to decrease (Fig 3). Female thrips begin to lay eggs on day 2 after emergence, and peak egg laying occurs on day 5 after emergence [38,39]. These results, together with the fact that adult female insects first detect their spawning sites through sensilla [40], suggest that *FintCSP1* is involved in finding oviposition sites. Additionally, male thrips begin mating on day 2 after emergence [39]. Therefore, the high level of *FintCSP1* expression in male thrips on day 2 might reflect the role of FintCSP1 in mate seeking behaviour. Therefore, FintCSP1 plays an important role in the chemoreception of *F*. *intonsa*.

Competitive binding methods have been employed to measure the binding affinity of CSPs to different odorants *in vitro* [41,42]. Here, we characterized the binding activity of FintCSP1 to *cis*-3-hexenal and its isomer *trans*-2-hexenal. The fluorescence binding assays showed that FintCSP1 specifically binds to *cis*-3-hexenal with high affinity (K_i_ of below 30 μM), whereas FintCSP1 could not bind to *trans*-2-hexenal, indicating that thrips can distinguish between different isomers (S2 Table). These results, together with the observation that OBP5 from *Apolygus lucorum* strongly binds to *cis*-3-hexenal [43], suggest that *cis*-3-hexenal is an essential odor molecule in the chemoreception of insects. Site-directed mutagenesis of specific amino acid residues can also elucidate the detailed interactions between CSPs and odor molecules [37,41]. Hydrophilic and hydrophobic residues contribute to the interaction between proteins and odor molecules [44]. Our homology modeling and molecular docking analyses predicted that four hydrophilic residues in FintCSP1 (Ser84, Lys26, Thr28, and Glu67) and one hydrophobic residue (Phe27) have important roles in binding and recognition of *cis*-3-hexenal (Fig 6C and S3 Table). These results, together with the individual mutations of Lys26, Thr28, and Glu67 to Ala that abolished the ability to bind *cis*-3-hexenal compared to the wild-type FintCSP1 (Fig 7B and Table 1), suggest that hydrophilic residues and Van der Walls interactions are involved in the binding of FintCSP1 to this ligand. Thus, we conclude that TZSV-infected pepper plants induce the emission of a specific plant volatile (*cis*-3-hexenal) that is subsequently recognized by FintCSP1, which increases the attractiveness of infected plants to thrips vectors.

Generally, insects use the total amount of VOCs in specific ratios and/or specific compounds to locate their host plants [11,45]. *Cis*-3-hexenal belongs to the C6-compound hexanal group of green leaf volatiles, which is biosynthesized from linolenic acid by various enzymatic reactions in response to abiotic or biotic stresses. Additionally, *cis*-3-hexenal is converted to *trans*-2-hexenal (leaf aldehyde) and *cis*-3-hexenol (leaf alcohol) via enzymatic catalysis [46,47]. *Cis*-3-hexenal is also a chemical cue for tobacco hornworm (*Manduca sexta*) and is a feeding stimulant for *M*. *sexta* larvae [48]. Our results showed that *cis*-3-hexenal emitted from TZSV-infected plants enhanced thrips attraction to the plants. Based on this finding, and together with the observation that TZSV-infected plants are more preferred by thrips vectors [7], we infer that *cis*-3-hexenal is a key chemical for the attraction of adult thrips. Furthermore, these results also strongly suggest a potential application of *cis*-3-hexenal in plant protection. Color traps and scents are commonly used to control thrips [49,50]. However, color traps also catch numerous natural enemies of thrips [51], making it necessary to add scents to enhance the trapping of thrips but not their natural predators. Although *cis*-3-hexenal has been shown to attract one of the planthoppers’ natural enemies, *A*. *nilaparvatae* [34], no studies have reported the effects of or the interaction between *cis*-3-hexenal and thrips natural enemies, such as *Orius* spp. (Heteroptera: Anthocoridae) [52]. Further investigations are needed to determine if *cis*-3-hexenal will pose a risk to *Orius* spp. populations, which would pave the way for the application of this attractant compound to fight thrips and their borne viruses.

## Materials and methods

### Insects, viruses, and plants

*Frankliniella intonsa* individuals were collected from the vegetable greenhouse of the Institute of Plant Protection, Fujian Academy of Agricultural Sciences, and confirmed based on morphology by Hongrui Zhang from the School of Plant Protection of Yunnan Agricultural University. *F*. *occidentalis* were originally donated by the Institute of Plant Protection, Hunan Academy of Agricultural Sciences, China. The insects were then reared on broad beans (*Vicia faba*) in an artificial climate incubator (PRX-250B, Saifu experimental instrument, Ningbo, China) (25°C; 65 ± 5% relative humidity; and a photoperiod of 14 h light/10 h darkness, light intensity: 4800 lx). Tomato (*Solanum lycopersicum*) fruits infected with tomato zonate spot virus (TZSV) were provided by the Institute of Biotechnology and Germplasm Resources, Yunnan Academy of Agricultural Sciences, China. The virus from tomato fruit was mechanically inoculated onto pepper (*Capsicum annuum*) leaves (30-day-old). Healthy pepper plants were obtained locally and were grown in a greenhouse maintained at 25°C under natural light conditions until they were used for virus transmission.

### Selection preference of thrips for host plants

*F*. *intonsa* and *F*. *occidentalis* preference tests of plant volatile cues were performed using a glass Y-tube olfactometer according to the method described by Wang et al. (2014) with minor modifications [8]. A Y-shaped tube (1-cm inner diameter) consisted of a central tube (10 cm in length) and two arms (10 cm in length, offset by 60°) connected to two different glass odor bottles. One glass odor bottle was 50 cm in height, with a 10-cm inner diameter that contained pepper plants (14-day post-viral infection); the other glass odor bottle was 5 cm in height, with a 3-cm inner diameter for plant VOCs. The airflow in the olfactometer (200 mL/min) was filtered through activated carbon and calibrated using a flow meter at the end of each arm. To remove all visual cues, the Y-tube was positioned horizontally in a black airtight cubic box (90 cm × 60 cm × 40 cm). The tested odor sources (pepper plant VOCs) were enclosed in the odor bottles 5 min prior to each test to allow the odors to fill the arms. Thrips (2-day-old, starved for 1 h) were individually placed in the central tube and allowed to choose between the two arms. When the thrips had climbed over 2/3 of the side arm and stayed there for more than 30 s, it was considered to have made a selection. If the thrips had not made a choice after being placed in the straight arm for 5 min, the insect behaviour was scored as “no selection,” and it was not counted in the total data. The direction of the Y-tube arms and the silicone tubes were adjusted after five tested thrips to eliminate the influence of position. The Y-tube was replaced after 10 tested thrips to eliminate the possible influence of existing odors on thrips behaviour. Each treatment consisted of 20 thrips, with three replicates per treatment.

### Expression analysis of olfactory genes in *F*. *intonsa* and *F*. *occidentalis* after exposure to TZSV-infected pepper plant odors

Fifty female *F*. *intonsa* or *F*. *occidentalis* individuals were placed in a clean and well-sealed glass bottle (45 mL, Cleman) containing leaves of TZSV-infected pepper plants (14-day post-viral infection) for 12 h. Glass bottles with mock-inoculated pepper plant leaves were used as a control. After 12 h of exposure, thrips were removed from the bottles and immediately frozen in liquid nitrogen for RNA extraction. Total RNA was isolated from 50 thrips with Trizol reagent (Ambion, Life Technologies, Carlsbad, CA, USA). First-strand cDNA was reverse transcribed from 1 μg of total RNA using the EasyScript Reverse Transcriptase Kit (Transgen Biotech, Beijing, China). *Fintβ-actin* (accession number: MT211604) and *Foccβ-actin* (accession number: XM_026432071.1) were selected as the reference genes; specific primers for *Fintβ-actin*, *Foccβ-actin*, *FoccCSP*, *FintCSP1*, *FintCSP2*, *FintOBP*, and *FintOR* were designed using Primer Premier 5.0 (S1 Table). Reverse transcription quantitative PCR (RT-qPCR) was performed using the GoTaq qPCR Master Mix (2X) kit (Promega, USA); each reaction was run as technical triplicates, while each sample was collected as biological triplicates. The expression changes of *FintCSP1*, *FintCSP2*, *FintOBP*, *FintOR*, or *FoccCSP* were calculated using the 2^−ΔΔCt^ method with normalization to *Fintβ-actin* or *Foccβ-actin*.

### Expression pattern of *FintCSP1* in antennae

The expression pattern of *FintCSP1* in antennae was investigated by RT-qPCR. Total RNA was isolated from 200 antennae of 1-day-old first-instar nymphs, 1-day-old second-instar nymphs, 1-day-old pupae, 2-day-old female adults, 5-day-old female adults, 10-day-old female adults, 2-day-old male adults, 5-day-old male adults, and 10-day-old male adults. Total RNA extraction, first-strand cDNA synthesis, and qPCR methods were as described above. *Fintβ-actin* was the reference gene; qPCR was conducted with specific primers for *Fintβ-actin* and *FintCSP1* (S1 Table). The relative transcript levels of *FintCSP1* in antennae at various differental stages were determined according to the 2^−ΔΔCt^ method. Relative transcript levels were normalized to those in 1-day-old first-instar nymphs, which were set to 1 [53].

### Immunofluorescence assays

To visualize FintCSP1 in *F*. *intonsa* antennae, female thrips antennae (2-day-old) were dissected under an optical microscope and fixed in 4% (w/v) paraformaldehyde (PFA) for 2 h and subsequently permeabilized with 2% (v/v) Triton X-100 (Sigma, USA) for 24 h at room temperature. The fixed samples were washed once with 100% ethanol and transferred to bleaching solution (100% ethanol: 30% H_2_O_2_; 2:1 [v/v]) for 2 h. After washing, the thrips were incubated with anti-mouse antibodies against FintCSP1 conjugated to fluorescein isothiocyanate (FITC) in phosphate buffered saline (PBS) containing 3% (w/v) bovine serum albumin (BSA) for 2 h at 37°C. The samples were washed three times with PBS, placed on a clean slide, and processed for immunofluorescence microscopy (Leica SP8, Germany).

### Transmission electron microscopy analysis

Female thrips (2-day-old) antennae were dissected, fixed, dehydrated, and embedded as described previously [54]. The samples were sectioned using an ultramicrotome (Leica UC5, Germany) and incubated with FintCSP1-specific IgG and immunogold-labeled anti-goat antibodies against rabbit IgG that had been conjugated with 12-nm-diameter gold particles (Jackson). The samples were observed under an electron microscope (Hitachi HT-7000, Japan).

### Bacterial expression and purification of FintCSP1 and FoccCSP

*FintCSP1* and *FoccCSP* DNA were released from pEASY-T1/*FintCSP1* and pEASY-T1/*FoccCSP*, respectively, by restriction digestion with *Bam*HI and *Xho*I and cloned into the pET-30a vector (Invitrogen). The resulting construct was transformed into Transetta (DE3) *Escherichia coli* competent cells (Transgen). The production of recombinant FintCSP1 and FoccCSP was induced by addition of isopropyl-β-D-1-thiogalactopyranoside (IPTG, 0.2 mM) at 15°C and purified by Ni ion affinity chromatography (Sangon Biotech, Shanghai, China). The target protein was verified by 15% SDS-PAGE, and its concentration was determined with a BCA protein assay kit (Thermo Scientific, Rockford, IL, USA). The recombinant purified protein was stored at −20°C until use.

### Fluorescence competitive binding assays

The binding affinity of FintCSP1 and FoccCSP to the fluorescent probe N-phenyl-1-naphthylamine (1-NPN) was tested using a Spectramax M2 fluorescence spectrometer (Molecular Devices, USA) as reported previously [55]. Volatile compounds and 1-NPN were first dissolved to a concentration of 1 mM in HPLC-grade methanol and dissolved to their indicated final concentration (2–20 μM) to titrate 2 μM protein solutions resuspended in 50 mM Tris-HCl (pH 7.4). The 1-NPN/FintCSP1 or 1-NPN/FoccCSP mixture was excited at 280 nm, and the emission spectra were recorded at 415 nm. The binding constants (K_d_) of FintCSP1 or FoccCSP to 1-NPN were calculated by Scatchard analysis using Prism 5 software (GraphPad, La Jolla, CA, USA). Assuming a protein activity of 100% and that the volatile compound binds to the protein in a 1:1 ratio at saturation, the dissociation constants (K_i_) of FintCSP1 or FoccCSP for each ligand were calculated according to the IC_50_ (the concentration when the ligand replaces 50% of the probe) value of the volatile compounds. The K_i_ values were calculated according to the following equation: K_i_ = [IC_50_]/(1 + [1-NPN]/K_1-NPN_), where [1-NPN] is the free concentration of 1-NPN, and K_1-NPN_ is the K_d_ of the 1-NPN/FintCSP1 or 1-NPN/FoccCSP.

If the K_i_ value was less than 20 μM, the tested ligands were considered to exhibit a strong binding affinity toward FintCSP1 or FoccCSP. A K_i_ value between 20 and 50 μM indicated that ligands have a medium binding affinity toward FintCSP1 or FoccCSP. A K_i_ value between 50 and 100 μM indicated that ligands have a weak binding affinity toward FintCSP1 or FoccCSP. K_i_ values that exceeded 100 μM indicated that ligands have no binding affinity to FintCSP1 or FoccCSP.

### RNA interference (RNAi) tests

The *FintCSP1*, *FintCSP2*, *FintOBP*, *FintOR*, *FoccCSP*, and enhanced green fluorescent protein (*EGFP*) coding sequences were obtained from GenBank under accession numbers MT199111, MT211602, OK067245, MT211603, AEP27186.1, and U55761.1, respectively. Primers were designed using Primer Premier 5.0, and the T7 promoter sequence (5′-TAATACGACTCACTATAGGG-3′) was added to the 5′ end of each primer (S1 Table). DNA fragments containing T7 promoter sequences were amplified by PCR using pEASY-T1/target gene or pUC-*EGFP* (preserved in the laboratory) plasmid DNA as template. Then, a double-stranded RNA (dsRNA) was produced from each target gene using the HiScribe T7 Quick High Yield RNA Synthesis Kit (New England Biolabs) according to the manufacturer’s instructions. Six hundred 2-day-old female *F*. *intonsa* or two hundred 2-day-old female *F*. *occidentalis* adults were anesthetized using CO_2_ and microinjected with 40 nL (3 μg/μL) dsRNA (ds*FintCSP1* ds*FintCSP2*, ds*FintOBP*, ds*FintOR* or ds*FoccCSP* or ds*EGFP*) using a CellTram Oil microinjector (Eppendorf, Hamburg, Germany). The injected thrips were kept on broad beans in an artificial climate incubator as described above and used in subsequent RNAi experiments. Three biological replicates were conducted for each treatment with each dsRNA.

### Electroantennogram (EAG) assays

At 24 h after dsRNA injection, the electrophysiological responses of *F*. *intonsa* antennae to six volatile components (i.e., m-cymene, *cis*-3-hexenal, (+)-2-carene, *trans*-2-hexenal, α-phellandrene, and α-humulene) or *F*. *occidentalis* antennae to *cis*-3-hexenal were recorded using EAG. Antennae of female adult thrips were cut off at the base, and the distal terminus was carefully removed. The tested compounds were dissolved in liquid paraffin, with pure liquid paraffin used as a negative control. An Ag-AgCl_2_ glass electrode and Kaissling electrode solution were used in the experiment according to Tian et al. [56]. The base of the antenna was inserted into the glass capillary filled with electrode solution at the end of the reference electrode; the top of the antenna was connected to the glass capillary at the end of the recording electrode to ensure no bubbles in the glass capillary. After successful connection, the antennae were stimulated with each chemical in the following order: liquid paraffin, volatile compounds, and liquid paraffin. Stimulus time was 0.2 s with each stimulus at 40 s intervals. At least 10 antennae were tested for each volatile compound. The EAG values to volatile compounds were calculated using the following equation: absolute value of EAG reaction = reaction value of the tested sample—average value of two control measurements.

### Homology modeling and molecular docking

The template structure of CSP was identified through NCBI BLAST and downloaded from the RCSB Protein Data Bank (http://www.rcsb.org/) as the PDB identifier 2GVS [57]. Homology modeling of FintCSP1 or FoccCSP was conducted with MOE v2018.01 [58]. The protonation state of the protein and the orientation of the hydrogens were optimized by LigX, at a pH of 7.0 and temperature of 300 K. First, the target sequence was aligned to the template sequence, and 10 independent intermediate models were built. These different homology models were the result of the permutational selection of different loop candidates and side-chain rotamers. Then, the intermediate model that scored best according to the GB/VI scoring function was chosen as the final model. The final model was subjected to further energy minimization using the AMBER10: EHT force field.

MOE Dock was used for molecular docking analysis of *cis*-3-hexenal on to FintCSP1 or FoccCSP. The 2D structure of *cis*-3-hexenal was downloaded from PubChem and converted to a 3D structure in MOE through energy minimization as ligand. The predicted structure of FintCSP1 or FoccCSP was used as the receptor. Prior to docking, the force field of AMBER10: EHT and the implicit solvation model of Reaction Field (R-field) were selected. The “induced fit” protocol was selected, in which the side chains of the binding site in the receptor are allowed to move according to ligand conformations, and a constraint was applied on their positions. The weight used for tethering side-chain atoms to their original positions was 10. First, all docked poses were ranked by the London dG scoring function, then force field refinement was applied on the top 30 poses, followed by a rescoring with the GBVI/WSA dG scoring function. The conformation with the lowest binding free energy was identified as the best probable binding mode, which was visualized by PyMOL (www.pymol.org).

### Site-directed mutagenesis

To verify the key amino acids of FintCSP1 and FoccCSP that bind to *cis*-3-hexenal, six amino acid residues of FintCSP1 were mutated (Lys26Ala, Phe27Ala, Thr28Ala, Glu67Ala, Ser84Ala, and the negative control residue Val132Ala) and nine amino acid residues of FoccCSP were mutated (Lys26Ala, Phe27Ala, Thr28Ala, Thr29Ala, Tyr31Ala, Asp64Ala, Glu67Ala, Gln87Ala, and the negative control residue Val132Ala) with the Fast Mutagenesis System Kit (Transgen Biotech, Beijing, China) using the pET-30a/FintCSP1 and pET-30a/FoccCSP plasmid DNA as template, respectively. Specific primers were designed using Primer Premier 5.0 according to the instructions of the Fast Mutagenesis System Kit (S1 Table). After validation by sequencing, each recombinant variant protein was produced and purified as described above, and their binding affinity to *cis*-3-hexenal was measured as described above.

### Statistical analyses

SPSS 21.0 software was used for data analysis (SPSS Inc., Chicago, IL, USA). The comparative analysis of *FintCSP1* expression levels in different stages, and EAG recordings of *F*. *intonsa* antennae in response to different doses of volatile compounds used one-way analysis of variance (ANOVA) followed by least significant difference (LSD) multiple comparison analysis. The comparative analysis of *FintCSP1*, *FintCSP2*, *FintOBP*, *FintOR*, and *FoccCSP* expression levels and EAG values among different treatment groups used *t*-tests. In the behavioural assays, the chi-squared (χ^2^) test was used for the olfactometry experiments. Competitive binding of wild-type FintCSP1, wild-type FoccCSP, and mutant variants with ligands were analyzed using Tukey’s honestly significant difference (HSD) test.

## Supporting information

S1 Fig TZSV-positive results using TZSV-N-specific primers. M: Marker, 1–19: *Frankliniella intonsa* collected from TZSV-infected pepper plants, 20: negative control using DEPC water, 21: positive control using TZSV-N plasmid.
(TIF)

S2 Fig Agarose gel electrophoresis of PCR product amplified from *Frankliniella intonsa* genomic DNA using specific *FintCSP1* primers. (TIF)

S3 Fig Nucleotide and deduced amino acid sequence of *FintCSP1* from *Frankliniella intonsa*. Predicted signal peptide sequence is underlined, and conserved cysteines are marked by red box. Stop codon is indicated with an asterisk.
(TIF)

S4 Fig Phylogenetic analysis of FintCSP1 from *Frankliniella intonsa* with other insects. Numbers on each branch are percentage support for the given branch, from 1,000 bootstrap replicates.
(TIF)

S5 Fig Ramachandran plot for FintCSP1 (A) and FoccCSP (B). Dark green dots represent residues in favored regions; yellow dots represent residues in allowed regions.
(TIF)

S6 Fig SDS-PAGE analysis of recombinant target proteins. M, Molecular weight marker; 1, non-induced pET30a/FoccCSP; 2, induced crude extract from pET30a/FoccCSP; 3, supernatant of pET30a/FoccCSP; 4, purified recombinant wild-type FoccCSP protein; 5−13, purified recombinant variants harboring the individual mutations Lys26Ala, Phe27Ala, Thr28Ala, Thr29Ala, Tyr31Ala, Asp64Ala, Glu67Ala, Gln87Ala, and Val132Ala, respectively.
(TIF)

S7 Fig Binding curve of 1-NPN to FoccCSP. Inset: Scatchard plot analysis. Data represent means of three independent replicates. Error bars indicate SE.
(TIF)

S8 Fig Homology modeling and molecular docking analysis for FoccCSP. (A) Structure-based sequence alignment between FoccCSP and the template (CSPsg4) structure. Identical or similar residues are highlighted in blue, and dissimilar ones are highlighted in red; darker colors indicate more similar or dissimilar residues. Residues corresponding to α-helix regions are marked by horizontal red lines; random coil or turn regions are marked by horizontal blue lines. Conserved cysteines are marked by orange box. (B) Predicted 3D model of FoccCSP (a) and superposed FoccCSP model onto the template structure (b). FoccCSP is shown in cyan, and the template structure is shown in violet. (C) Interaction diagram between FoccCSP amino acid residues and *cis*-3-hexenal. *Cis*-3-hexenal is shown in cyan. Surrounding residues in the binding pocket are colored in yellow. Hydrogen bond is depicted as a green dashed line.
(TIF)

S1 Table Primers used in this study. (DOCX)

S2 Table Binding assays of recombinant FintCSP1 against *cis*-3-hexenal and *trans*-2-hexenal. (DOCX)

S3 Table Docking scores and interactions between FintCSP1 and *cis*-3-hexenal. (DOCX)

S4 Table Docking scores and interactions between FoccCSP and *cis*-3-hexenal. (DOCX)
