# Supplementary figures and images for: Chemosensory protein regulates the behavioural response of Frankliniella intonsa and Frankliniella occidentalis to tomato zonate spot virus–Infected pepper (Capsicum annuum)
Source: PLoS Pathog. 2023 May 8;19(5):e1011380. doi: 10.1371/journal.ppat.1011380 (PMC10194981; doi:10.1371/journal.ppat.1011380)

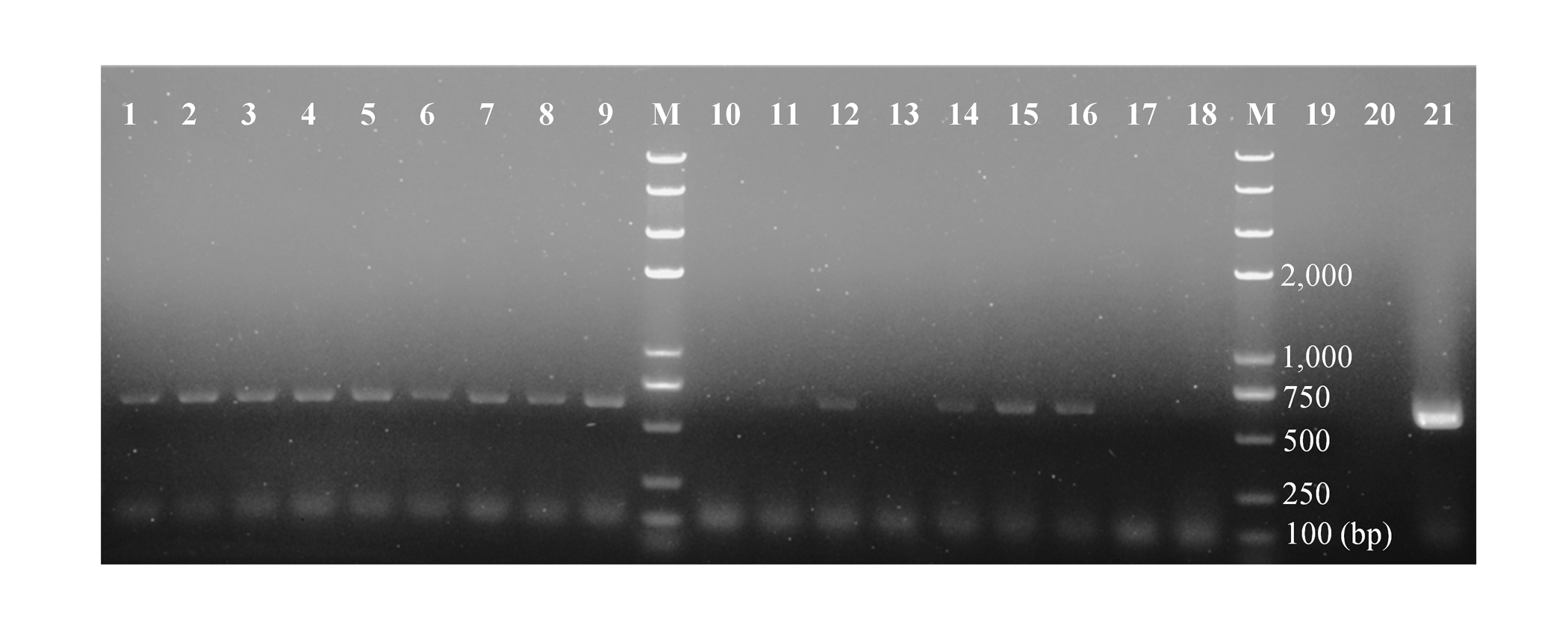

Supplement: S1 Fig — M: Marker, 1–19: Frankliniella intonsa collected from TZSV-infected pepper plants, 20: negative control using DEPC water, 21: positive control using TZSV-N plasmid. (TIF) [file ppat.1011380.s001.tif]

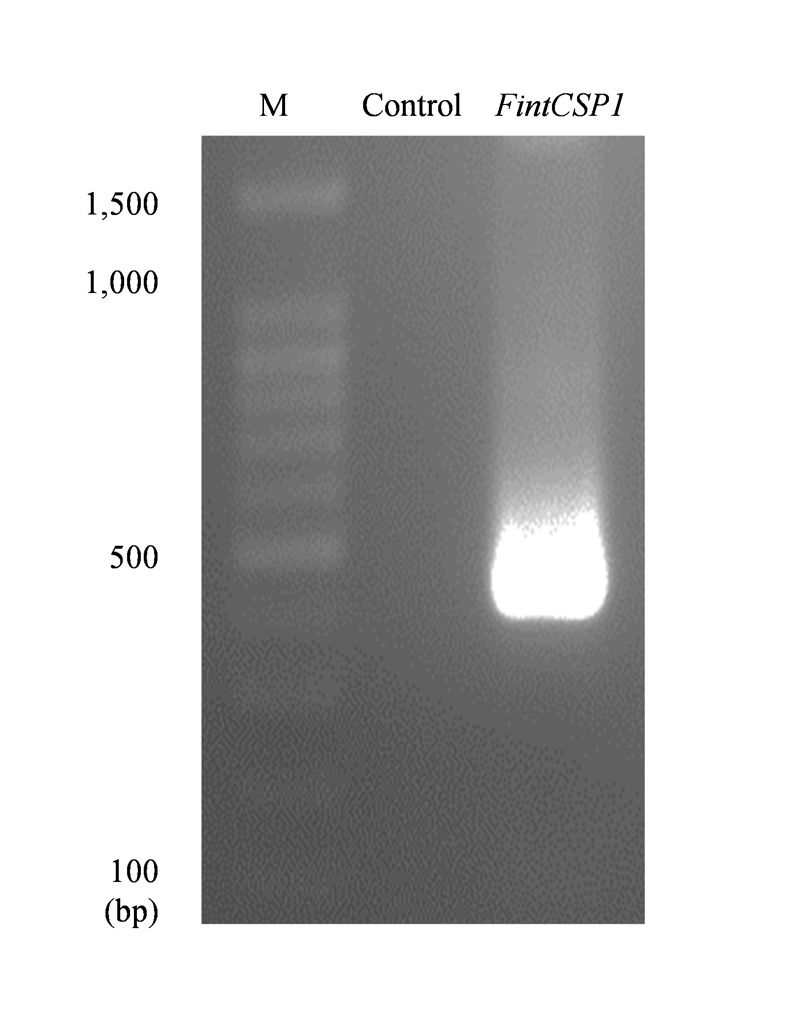

Supplement: S2 Fig — (TIF) [file ppat.1011380.s002.tif]

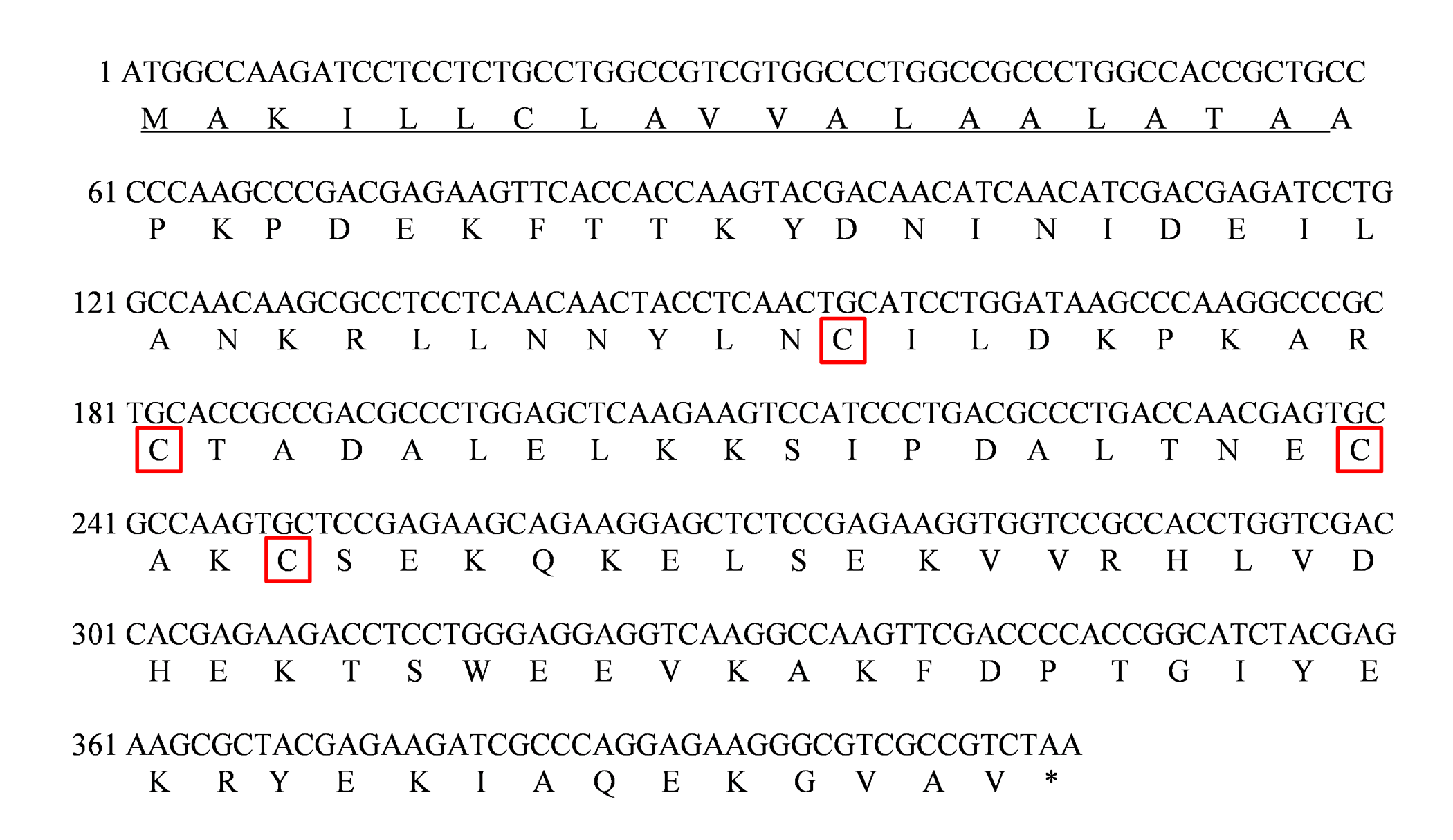

Supplement: S3 Fig — Predicted signal peptide sequence is underlined, and conserved cysteines are marked by red box. Stop codon is indicated with an asterisk. (TIF) [file ppat.1011380.s003.tif]

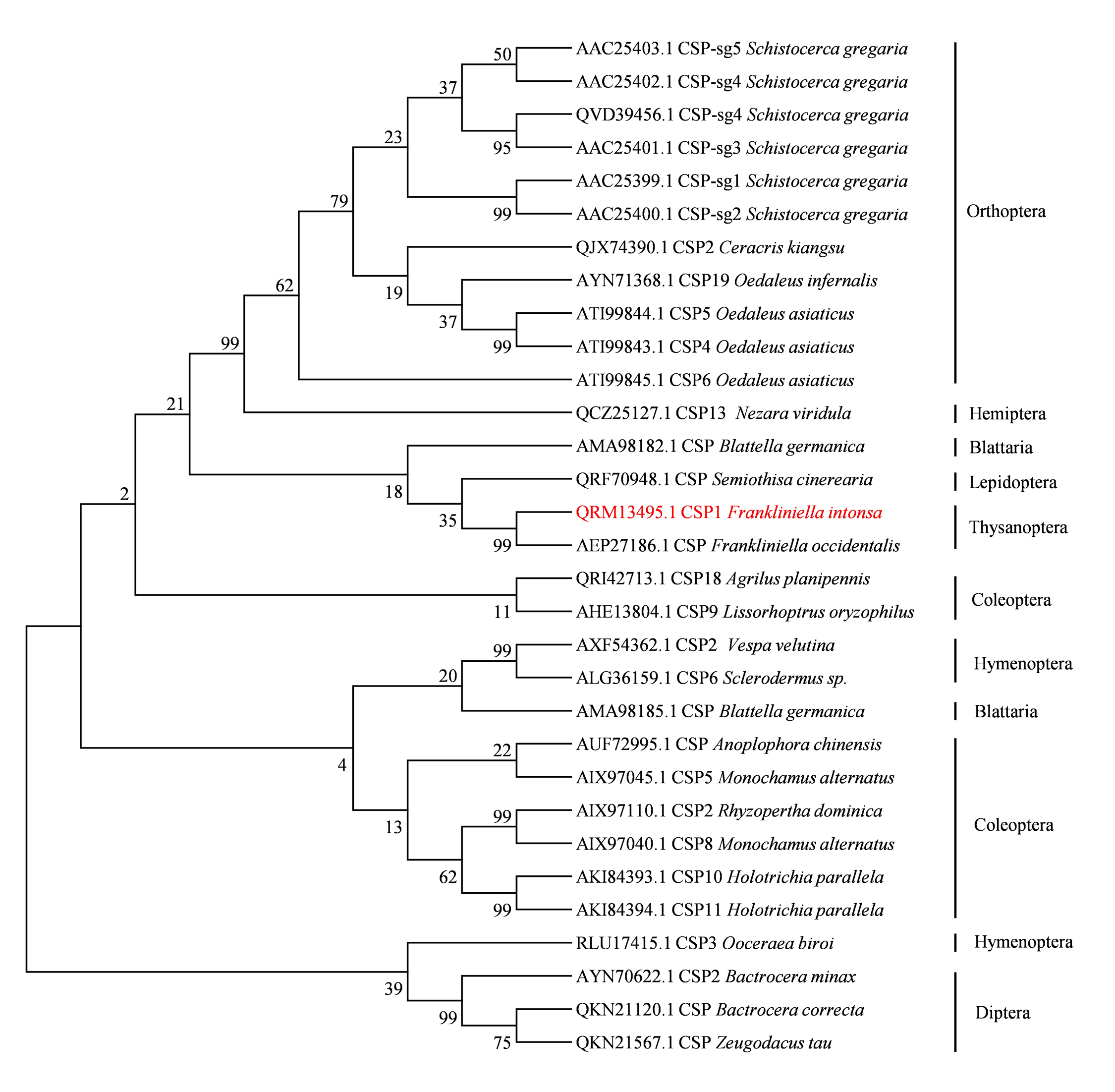

Supplement: S4 Fig — Numbers on each branch are percentage support for the given branch, from 1,000 bootstrap replicates. (TIF) [file ppat.1011380.s004.tif]

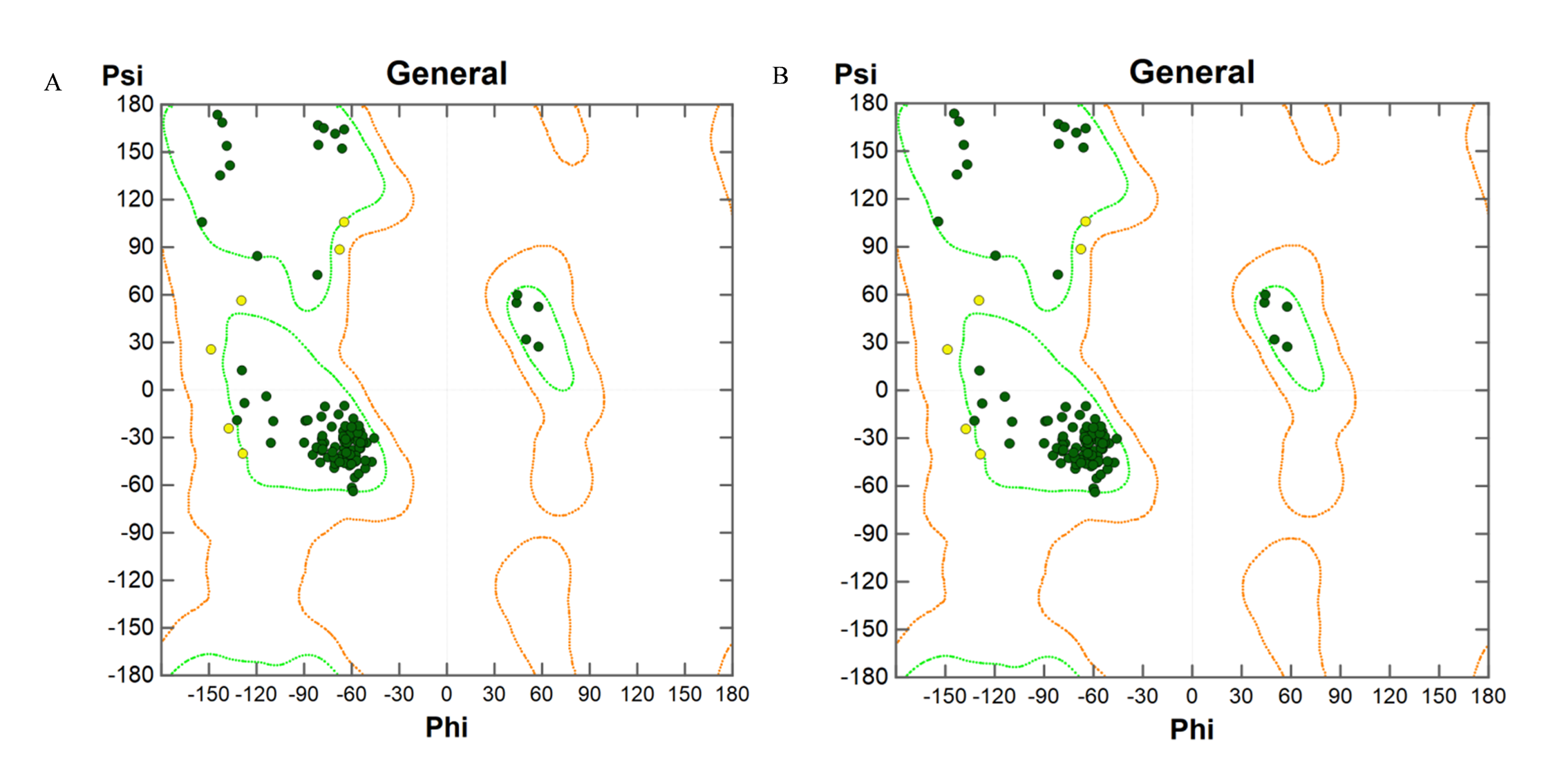

Supplement: S5 Fig — Ramachandran plot for FintCSP1 (A) and FoccCSP (B). Dark green dots represent residues in favored regions; yellow dots represent residues in allowed regions. (TIF) [file ppat.1011380.s005.tif]

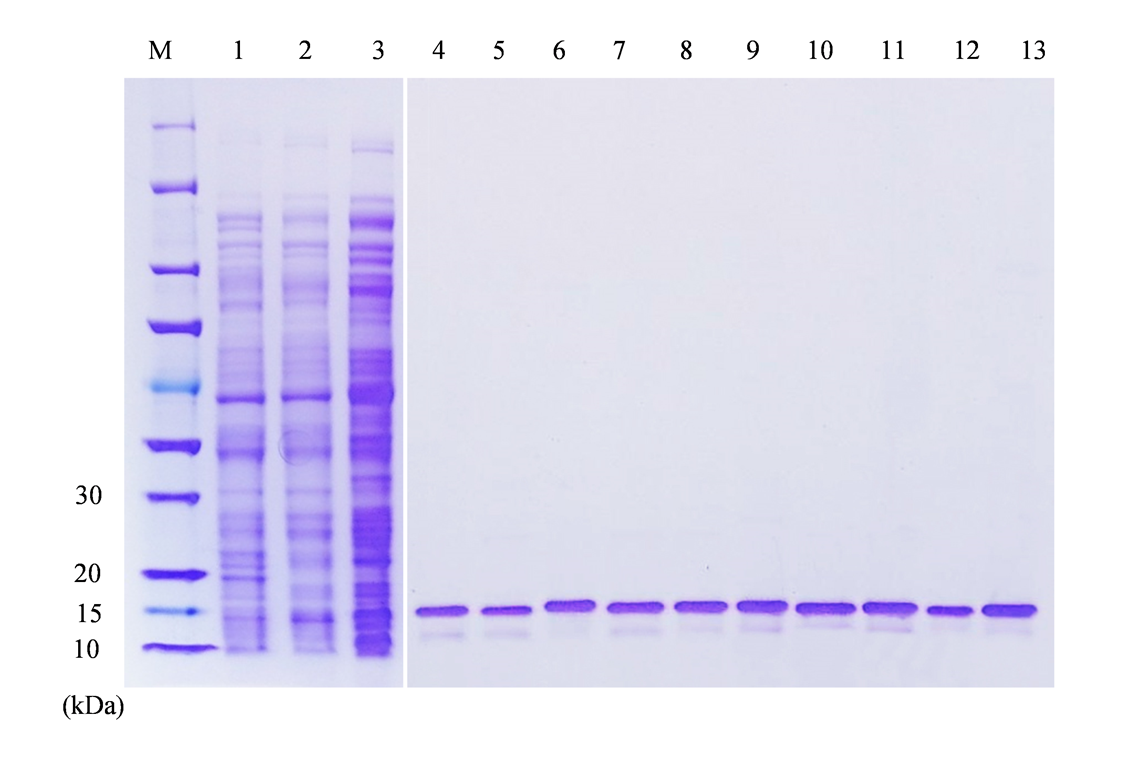

Supplement: S6 Fig — M, Molecular weight marker; 1, non-induced pET30a/FoccCSP; 2, induced crude extract from pET30a/FoccCSP; 3, supernatant of pET30a/FoccCSP; 4, purified recombinant wild-type FoccCSP protein; 5−13, purified recombinant variants harboring the individual mutations Lys26Ala, Phe27Ala, Thr28Ala, Thr29Ala, Tyr31Ala, Asp64Ala, Glu67Ala, Gln87Ala, and Val132Ala, respectively. (TIF) [file ppat.1011380.s006.tif]

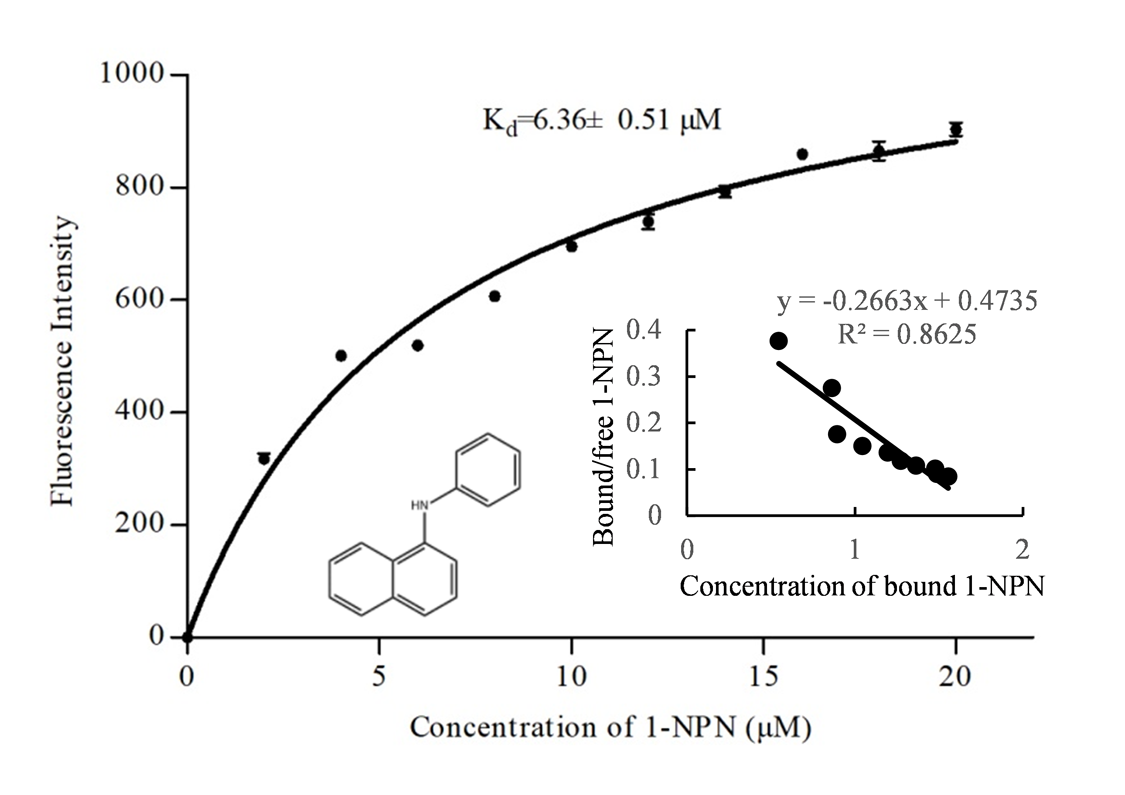

Supplement: S7 Fig — Inset: Scatchard plot analysis. Data represent means of three independent replicates. Error bars indicate SE. (TIF) [file ppat.1011380.s007.tif]

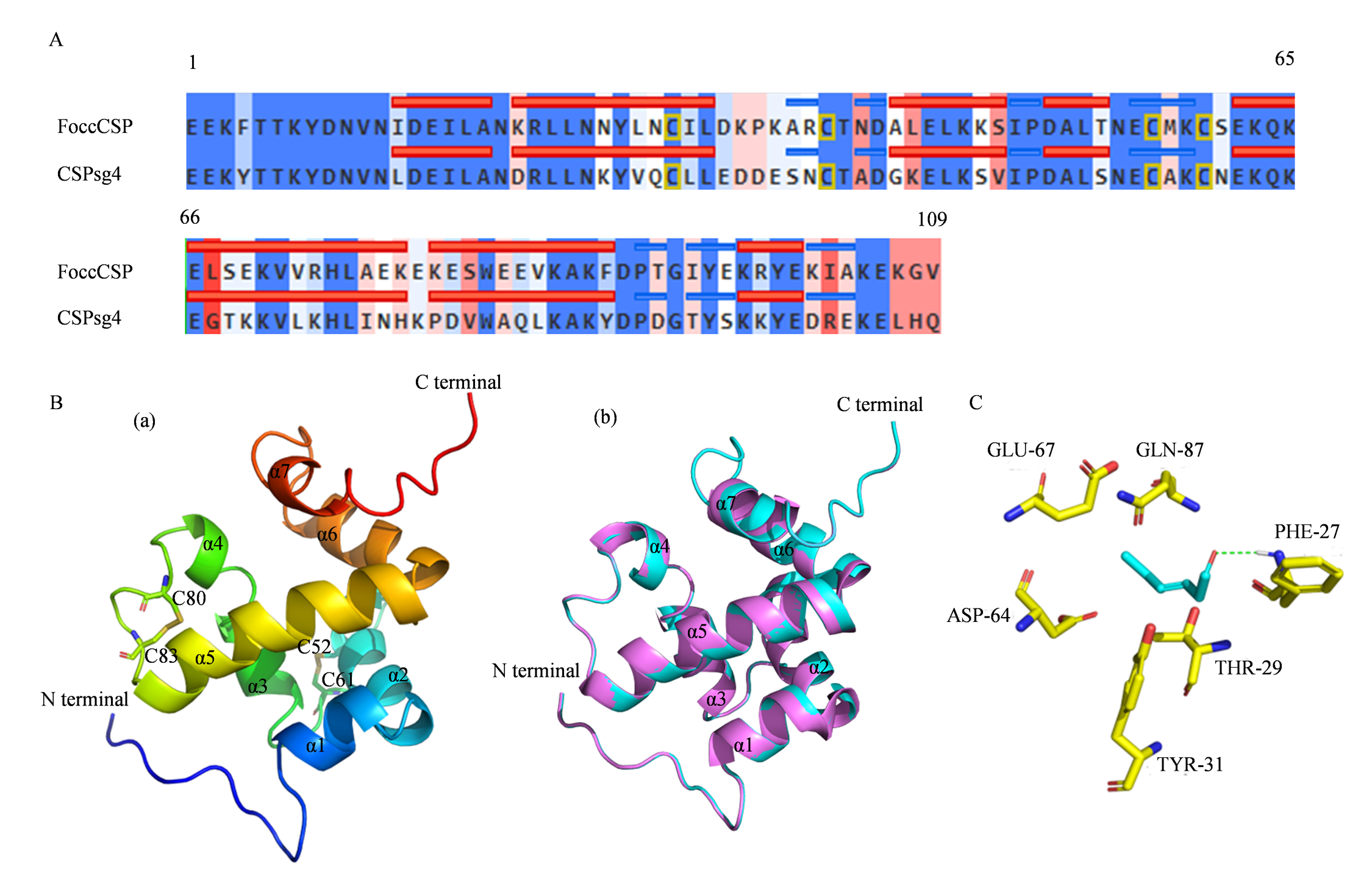

Supplement: S8 Fig — (A) Structure-based sequence alignment between FoccCSP and the template (CSPsg4) structure. Identical or similar residues are highlighted in blue, and dissimilar ones are highlighted in red; darker colors indicate more similar or dissimilar residues. Residues corresponding to α-helix regions are marked by horizontal red lines; random coil or turn regions are marked by horizontal blue lines. Conserved cysteines are marked by orange box. (B) Predicted 3D model of FoccCSP (a) and superposed FoccCSP model onto the template structure (b). FoccCSP is shown in cyan, and the template structure is shown in violet. (C) Interaction diagram between FoccCSP amino acid residues and cis-3-hexenal. Cis-3-hexenal is shown in cyan. Surrounding residues in the binding pocket are colored in yellow. Hydrogen bond is depicted as a green dashed line. (TIF) [file ppat.1011380.s008.tif]
