# Supplementary material for: Chemosensory protein regulates the behavioural response of Frankliniella intonsa and Frankliniella occidentalis to tomato zonate spot virus–Infected pepper (Capsicum annuum)
Source: PLoS Pathog. 2023 May 8;19(5):e1011380. doi: 10.1371/journal.ppat.1011380 (PMC10194981; doi:10.1371/journal.ppat.1011380)
Supplement: S1 Table — (DOCX) [file ppat.1011380.s009.docx]

**S1 Table.** Primers used in this study

| Primer name | | Sequence (5′-3′) |
| --- | --- | --- |
| For RT-PCR | |  |
| *FintCSP1-F* | | CGGGATCCATGGCCAAGATCCTCCTCTGC |
| *FintCSP1-R* | | CCCTCGAGTTAGACGGCGACGCCCTTCTC |
| *FoccCSP-F* | | CGGGATCC ATGGCCAAGATCCTCC |
| *FoccCSP-R* | | CCCTCGAG TCAGACAGCGACTCCC |
| *TZSV-N-F* | | AAAGATTCAAGAACTATTGGCT |
| *TZSV-N-R* | | TCTCAGTGAACTCCACGCTA |
| For RT-qPCR | |  |
| *qFintβ-actin-*F | | GCATACACGAAACGACCTACAACTC |
| *qFintβ-actin-*R | | GGTAGTACCACCCGACAATACGG |
| *qFintCSP1-*F | | TCAAGAAGTCCATCCCTGACGC |
| *qFintCSP1-*R | | GGTGCCGATGAAGAGCAGACC |
| *qFintCSP2-*F | | TGGGAGCGTCTCACCAACAA |
| *qFintCSP2-*R | | AACAAATTACATAGCCAGTGCATCAC |
| *qFintOBP-*F | | TGATGGTGCTGAAGAACAACAAGGTG |
| *qFintOBP-*R | | CGAGGTGAAGGCGGCGATGACT |
| *qFintOR-*F | | AATGGAGCAGCCAACCA |
| *qFintOR-*R | | GGATAACAAATGCCAGAAGG |
| *qFoccβ-actin-F* | | CACCACCGCTGAGCGTGAAATCG |
| *qFoccβ-actin-R* | | GTGATGACCTGACCGTCGGGAAGC |
| *qFoccCSP-F* | | GAAGGAGCTGTCCGAGAAGG |
| *qFoccCSP-R* | | CAGCGACTCCCTTCTCCTTG |
| For dsRNA synthesis | | |
| ds*EGFP-*F | TAATACGACTCACTATAGGGCGAGGAGCTGTTCACCGG | |
| ds*EGFP-*R | TAATACGACTCACTATAGGGTCCTCGATGTTGTGGCGG | |
| ds*FintCSP1-*F | TAATACGACTCACTATAGGGAGTTCACCACCAAGTACGACAACATCA | |
| ds*FintCSP1-*R | TAATACGACTCACTATAGGGACGGCGACGCCCTTCTCCTG | |
| ds*FintCSP2-*F | TAATACGACTCACTATAGGGGTGCTCGTGCTCGCCGAAGC | |
| ds*FintCSP2-*R | TAATACGACTCACTATAGGGGTGATCTGGGTCATACTTGTTGGT | |
| ds*FintOBP-*F | TAATACGACTCACTATAGGGATGGCGCTGTGCCACCC | |
| ds*FintOBP-*R | TAATACGACTCACTATAGGGGGTGAAGGCGGCGATGACTC | |
| ds*FintOR-*F | TAATACGACTCACTATAGGGTTGGTAGCTTGTCGGAGTG | |
| ds*FintOR-*R | TAATACGACTCACTATAGGGGCAGTTGCCTTGGGATG | |
| *dsFoccCSP-F* | TAATACGACTCACTATAGGG TCAACATCGACGAGATCCTG | |
| *dsFoccCSP-R* | TAATACGACTCACTATAGGG CTTGGCGATCTTCTCGTACC | |
| For site-directed mutagenesis | | |
| FintCSP1*-*Lys26Ala-F | | CCCAAGCCCGACGAGGCGTTCACCACCAAG |
| FintCSP1*-*Lys26Ala-R | | GCCTCGTCGGGCTTGGGGGCAGCGGTGG |
| FintCSP1-Phe27Ala-F | | AAGCCCGACGAGAAGGCCACCACCAAGTAC |
| FintCSP1-Phe27Ala-R | | GCCTTCTCGTCGGGCTTGGGGGCAGCGGTG |
| FintCSP1-Thr28Ala-F | | AAGCCCGACGAGAAGTTCGCCACCAAGTACG |
| FintCSP1-Thr28Ala-R | | CGAACTTCTCGTCGGGCTTGGGGGCAGCGGTG |
| FintCSP1-Glu67Ala-F | | CACCGCCGACGCCCTGGCGCTCAAGAAGTCCA |
| FintCSP1-Glu67Ala-R | | GCCAGGGCGTCGGCGGTGCAGCGGGCCT |
| FintCSP1-Ser84Ala-F | | GAGTGCGCCAAGTGCGCCGAGAAGCAGA |
| FintCSP1-Ser84Ala-R | | CGCACTTGGCGCACTCGTTGGTCAGGGC |
| FintCSP1-Val132Ala-F | | CCCAGGAGAAGGGCGCCGCCGTCTAA |
| FintCSP1-Val132Ala-R | | GCGCCCTTCTCCTGGGCGATCTTCTCG |
| FoccCSP-Lys26Ala-F | | CCCAAGCCCGAGGAGGCGTTCACCACC |
| FoccCSP-Lys26Ala-R | | GCCTCCTCGGGCTTGGGGGCGGCGGCG |
| FoccCSP-Phe27Ala-F | | CCAAGCCCGAGGAGAAGGCCACCACCAAGT |
| FoccCSP-Phe27Ala-R | | GCCTTCTCCTCGGGCTTGGGGGCGGCG |
| FoccCSP-Thr28Ala-F | | GCCCGAGGAGAAGTTCGCCACCAAGTACG |
| FoccCSP-Thr28Ala-R | | CGAACTTCTCCTCGGGCTTGGGGGCG |
| FoccCSP-Thr29Ala-F | | CCCGAGGAGAAGTTCACCGCCAAGTACGACA |
| FoccCSP-Thr29Ala-R | | CGGTGAACTTCTCCTCGGGCTTGGGG |
| FoccCSP-Tyr31Ala-F | | GGAGAAGTTCACCACCAAGGCCGACAACGTC |
| FoccCSP-Tyr31Ala-R | | GCCTTGGTGGTGAACTTCTCCTCGGGC |
| FoccCSP-Asp64Ala-F | | CCCGCTGCACCAACGCCGCTCTGGAG |
| FoccCSP-Asp64Ala-R | | GCGTTGGTGCAGCGGGCCTTGGGCTT |
| FoccCSP-Glu67Ala-F | | GCACCAACGACGCTCTGGCGCTCAAGAAA |
| FoccCSP-Glu67Ala-R | | GCCAGAGCGTCGTTGGTGCAGCGGGC |
| FoccCSP-Gln87Ala-F | | TGAAGTGCTCGGAGAAGGCGAAGGAGCTGT |
| FoccCSP-Gln87Ala-R | | GCCTTCTCCGAGCACTTCATGCACTCGTTGG |
| FoccCSP-Val132Ala-F | | GCCAAGGAGAAGGGAGCCGCTGTCTGA |
| FoccCSP-Val132Ala-R | | GCTCCCTTCTCCTTGGCGATCTTCTCGTACCTCT |

Note: Red letters indicate the restriction sites, and the underlined position is the T7 promoter sequence.
