# Supplementary material for: Chemosensory protein regulates the behavioural response of Frankliniella intonsa and Frankliniella occidentalis to tomato zonate spot virus–Infected pepper (Capsicum annuum)
Source: PLoS Pathog. 2023 May 8;19(5):e1011380. doi: 10.1371/journal.ppat.1011380 (PMC10194981; doi:10.1371/journal.ppat.1011380)
Supplement: S2 Table — (DOCX) [file ppat.1011380.s010.docx]

**S2 Table.** Binding assays of recombinant FintCSP1 against *cis*-3-hexenal and *trans*-2-hexenal

| Ligand | CAS no. | IC_50_（μM） | K_i_（μM） |
| --- | --- | --- | --- |
| *cis*-3-hexenal | 6789-80-6 | 36.15±1.0 | 28.68±0.79 |
| *trans*-2-hexenal | 6728-26-3 | nd | nd |

Affinities are given as means ± SE. IC_50_, ligand concentration displacing 50% of the fluorescence intensity of the FintCSP1/1-NPN complex; K_i_, dissociation constant; nd, not determined (binding constant was not calculated; IC_50_ > 100 μM).
