# Supplementary material for: Chemosensory protein regulates the behavioural response of Frankliniella intonsa and Frankliniella occidentalis to tomato zonate spot virus–Infected pepper (Capsicum annuum)
Source: PLoS Pathog. 2023 May 8;19(5):e1011380. doi: 10.1371/journal.ppat.1011380 (PMC10194981; doi:10.1371/journal.ppat.1011380)
Supplement: S3 Table — (DOCX) [file ppat.1011380.s011.docx]

**S3 Table.** Docking scores and interactions between FintCSP1 and *cis*-3-hexenal

| Ligand | Score (kcal/mol) | No. of HBs | No. of π interaction pairs | No. of VDW interactions | Residues involved |
| --- | --- | --- | --- | --- | --- |
| *cis*-3-hexenal | –4.2759 | 1 | – | 4 | K26, F27, T28, E67, S84 |

HB, hydrogen bond; VDW, Van Der Waals.
